# De novo assembly and comparative analysis of the transcriptome of embryogenic callus formation in bread wheat (*Triticum aestivum* L.)

**DOI:** 10.1186/s12870-017-1204-2

**Published:** 2017-12-19

**Authors:** Zongli Chu, Junying Chen, Junyan Sun, Zhongdong Dong, Xia Yang, Ying Wang, Haixia Xu, Xiaoke Zhang, Feng Chen, Dangqun Cui

**Affiliations:** 1 Agronomy College/Collaborative Innovation Center of Henan Grain Crops/National Key Laboratory of Wheat and Maize Crop Science, Henan Agricultural University, 15 Longzihu College District, Zhengzhou, 450046 People’s Republic of China; 2 Xinyang Agriculture and Forestry University, Xinyang, 464000 China; 3 Agronomy College, North West Agriculture and Forestry University, Yangling, 712100 China

**Keywords:** Transcriptome, Embryo culture, Embryogenic callus, Immature embryo, Mature embryo, Wheat (*Triticum aestivum* L.)

## Abstract

**Background:**

During asexual reproduction the embryogenic callus can differentiate into a new plantlet, offering great potential for fostering in vitro culture efficiency in plants. The immature embryos (IMEs) of wheat (*Triticum aestivum* L.) are more easily able to generate embryogenic callus than mature embryos (MEs). To understand the molecular process of embryogenic callus formation in wheat, de novo transcriptome sequencing was used to generate transcriptome sequences from calli derived from IMEs and MEs after 3d, 6d, or 15d of culture (DC).

**Results:**

In total, 155 million high quality paired-end reads were obtained from the 6 cDNA libraries. Our de novo assembly generated 142,221 unigenes, of which 59,976 (42.17%) were annotated with a significant Blastx against nr, Pfam, Swissprot, KOG, KEGG, GO and COG/KOG databases. Comparative transcriptome analysis indicated that a total of 5194 differentially expressed genes (DEGs) were identified in the comparisons of IME vs. ME at the three stages, including 3181, 2085 and 1468 DEGs at 3, 6 and 15 DC, respectively. Of them, 283 overlapped in all the three comparisons. Furthermore, 4731 DEGs were identified in the comparisons between stages in IMEs and MEs. Functional analysis revealed that 271transcription factor (TF) genes (10 overlapped in all 3 comparisons of IME vs. ME) and 346 somatic embryogenesis related genes (SSEGs; 35 overlapped in all 3 comparisons of IME vs. ME) were differentially expressed in at least one comparison of IME vs. ME. In addition, of the 283 overlapped DEGs in the 3 comparisons of IME vs. ME, excluding the SSEGs and TFs, 39 possessed a higher rate of involvement in biological processes relating to response to stimuli, in multi-organism processes, reproductive processes and reproduction. Furthermore, 7 were simultaneously differentially expressed in the 2 comparisons between the stages in IMEs, but not MEs, suggesting that they may be related to embryogenic callus formation. The expression levels of genes, which were validated by qRT-PCR, showed a high correlation with the RNA-seq value.

**Conclusions:**

This study provides new insights into the role of the transcriptome in embryogenic callus formation in wheat, and will serve as a valuable resource for further studies addressing embryogenic callus formation in plants.

**Electronic supplementary material:**

The online version of this article (10.1186/s12870-017-1204-2) contains supplementary material, which is available to authorized users.

## Background

Efficient plant regeneration from in vitro cultured cells and tissues is a prerequisite requirement for the successful application of plant genetic engineering, which has been incorporated into the improvement and breeding of many crops [1]. However, the regeneration efficiency of a great number of plant species is still low under in vitro conditions. Embryogenic callus formation offers great potential for fostering in vitro culture efficiency whereby somatic cells are induced to differentiate embryogenic cells and form somatic embryos that develop into new plants [2, 3]. The ability to initiate embryogenic cultures is controlled by the intrinsic and external factors of the explants, including the genotype, the developmental stage, and the different tissue of the explants, as well as the factors associated with the medium as perceived by the complementary sensors in cells, including stress, phytohormones, and the artificial nutritional environment in the culture process [4–6]. In plants, the tissue culture response (TCR), regenerative property, regeneration response, and somatic embryogenesis are all traits that are used to study tissue culture.

To understand the molecular mechanisms of gene regulation in in vitro culture, the expression of the somatic embryogenesis-related genes and signaling regulation has been researched in many species, including *Cyclamen persicum* [7], oil palm [8], cotton [9, 10], Arabidopsis [11], soybean [12] and chicory [13], using different approaches including cDNA microarray, in situ hybridization (ISH), and suppression subtractive hybridization (SSH). Transcriptional profiling has shown that many genes showing differential expression during somatic embryogenesis (SE) in cereals may be associated with embryogenic callus formation. Some transcription factor (TF) genes such as the LEC genes, including *Arabidopsis* LEC1 and L1 L, encode HAP3-related transcription factors [14–16], while *Arabidopsis* LEC2, FUS3, and ABI3, as well as maize Viviparous1 (Vp1), encode the B3 domain transcription [17, 18]. Some members of the AP2/ERF family [19] encoded by BBM [20, 21], AGL15 [22], and WUSCHEL (WUS) genes [23], are essential for maintaining embryogenic competency of plant somatic cells.

The bread wheat (*Triticum aestivum* L.) is one of the most important human food crops. Considerable effort and progress has been made in tissue culture system optimization over the last few decades, however tissue culture efficiency is still low and lags behind that of other plants such as rice and maize [24, 25]. A number of studies on wheat embryogenic callus formation-associated genes have been performed using Northern and semi qRT-PCR analyses [26, 27]. Considerable data have been accumulated regarding the influence upon TCR, but with a limited knowledge of the genes involved therein.

Next-generation sequencing (NGS) technology has high sensitivity and includes both low- and high-level gene expression [28–30], and has been increasingly applied in numerous plants to elucidate development [31], senescence [32, 33], effects of different factors on plant growth [32, 33], and determine transcriptome changes in response to various abiotic stresses such as cold [34], salt [35], drought [36], water [37], and has also been used in wheat transcriptome studies regarding response to stress or disease [38, 39]. In terms of in vitro culturing in plants, NGS has been successfully used in cotton [40], *Picea balfouriana* [41], camphor tree [42], Maize [43], and in Ramie [44] for transcriptome analysis. However, few reports have been found regarding the transcriptome in wheat in vitro culture at the genomic scale. Thus, the study of gene expression patterns and functioning during callus formation could provide a molecular basis for embryogenic callus formation in wheat. However, the large size and polyploid complexity of the wheat genome [45], and the difficulties of tissue culture, have been obstacles to the study of the genes involved in wheat embryogenic callus formation. Using Illumina deep sequencing, we compared the transcriptome expression changes between IMEs and MEs for in vitro culture at 3d, 6d, and 15d, and between developmental stages in IMEs and MEs, combined with the functional annotation of the DEGs, especially of those that are potentially involved in embryogenic callus formation. This will provide novel insights into wheat embryogenic callus formation and the transcriptome data presented here will serve as a valuable resource for future studies of the genes and gene regions involved.

## Methods

### Plant material and tissue culture

The bread wheat cultivar Zhoumai 18 was grown in the Zhengzhou experimental field (longitude E 113.65°, latitude N 34.76°) of the Henan Agricultural University of China during the 2011–2012 cropping season. Harvested seeds were planted in the same experimental field in October 5 2012, after which they were transplanted into a greenhouse in January 15 2013 under the following controlled environmental conditions: 75% relative humidity, 26/20 °C day/night temperature, 12−/12-h light/dark photoperiod, and a light intensity of 10,000 lx. Anthesis was recorded in February of 2013 when 50% of the plants had reached the flowering stage.

Immature seeds from the main spikes were harvested 14 days after pollination. The mature seeds were soaked in running tap water for 4 h. Immature and mature seeds were surface-sterilized for 30 s in 75% (*v*/v) ethanol, followed by 6 min immersion in 0.1% (m/v) mercuric chloride solution with agitation, and then rinsed 4 times with sterilized distilled water. The IMEs and MEs were extracted from the sterilized seeds on a clean bench and explants were placed with the scutellum upwards in sterile Petri dishes containing solid Murashige and Skoog (MS) medium (MS basal salts, Gamborg’s B5 vitamins, and 2 mg L^−1^ 2, 4-Dichlorophenoxyacetic acid). They were then grown in a growth chamber at 22–24 °C in the dark. The MEs and IMEs were cultured in 3 biological replicates, each replicate consisting of 15 plates and each plate containing 10 embryos. The embryos were harvested at 3 DC, 6 DC, and 15 DC from 5 plates in each of the 3 replicates, after which they were snap-frozen in liquid nitrogen and stored at −80 °C until RNA extraction.

### RNA extraction, RNA-seq library construction and Illumina sequencing

Total RNA was extracted from the IME and ME callus in the 3 biological replicates using TRIzol reagent (Invitrogen, Carlsbad, CA, USA) according to the manufacturer’s instructions, and then characterized on a 1% agarose gel. The purity of the RNA was examined using a NanoDrop 2000 spectrophotometer (IMPLEN, CA, USA), and the integrity was examined using an Agilent 2100 Bioanalyzer (Agilent Technologies, CA, USA). The RNA samples of the 3 biological replicates were mixed in equal amounts and used for the construction of libraries by the Biomarker Biotechnology Corporation (Beijing, China). A total of 3 μg RNA for each sample was used as input material for cDNA library construction. The mRNA was enriched and purified with oligo (dT)-rich magnetic beads and then broken into short fragments. Taking these cleaved mRNA fragments as templates, first strand cDNA was synthesized and primed by oligo-dT and the second strand cDNA was synthesized using random primers. The resulting cDNAs were then subjected to end-repair and phosphorylation using T4 DNA polymerase and Klenow DNA polymerase.

Following this, an “A” base was inserted as an overhang at the 3′ ends of the repaired cDNA fragments and Illumina paired-end solexa adaptors were subsequently ligated to these cDNA fragments to distinguish the different sequencing samples. To select a size range of templates for downstream enrichment, the products of the ligation reaction were purified and visualized on a 2% agarose gel. Next, PCR amplification was performed to enrich the purified cDNA template.

RNA sequencing was performed on an Illumina HiSeq (TM) 2000 (San Diego, CA, USA). The RNA-seq read data were deposited in the NCBI Sequence Read Archive (SRP093588).

### De novo assembly and functional annotation

Clean, high quality reads were obtained after filtering the adaptor sequences and reads with ambiguous “N” bases and with a base quality less than Q30 (and more than 10% base quality less than Q20). The clean reads were assembled into contigs using the Trinity method (Grabherr MG, et al., 2011) using an optimized k-mer length (25-mer). Subsequently, the contigs were assembled into transcripts according to the paired-end information, after which they were clustered based on sequence similarity. Finally, the longest transcript in each cluster was regarded as a unigene, and the singletons were combined together as total unigenes. All unigenes were mapped to the reference sequences (wheat unigenes from NCBI) and allowed no more than one nucleotide mismatch. For annotation, all unigenes were used for the blast search and annotation against a series of public databases using BLASTx (E-value–5 ≤ 10), including the NCBI non-redundant (Nr) protein databases (http://www.ncbi.nlm.nih.gov), the Swiss-Prot protein database (http://www.expasy.ch/sprot), the Kyoto Encyclopedia of Genes and Genomes (KEGG) pathway database (http://www.genome.jp/kegg), the Cluster of Orthologous Groups (COG) database (http://www.ncbi.nlm.nih.gov/COG), and the pfam database. Functional classification by gene ontologies (GO) of all unigenes was performed using Blast2GO software.

### Identification of differentially expressed genes

To quantify the gene expression level, the number of mapped clean reads was estimated using RSEM [46] for each sample, and the normalized expression values RPKM (reads per kilo base of exon model per million mapped reads) of each unigene in the 6 libraries were used as the value of gene expression levels [47]. To determine significant differences in gene expression, we used threshold criteria based on the FDR statistical method and compared the normalized expression values RPKM using a threshold value of *P* ≤ 0.001 and |log2 Ratio| ≥ 1 based on the FDR < 0.05.

### Analysis of transcription factors

Putative transcription factors were identified by BLASTx against the Plant Transcription Factor Database (PlnTFDB) 3.0 [48] using the bread wheat Transcription Factor (http://planttfdb.cbi.pku.edu.cn/index.php?sp=Tae), *Arabidopsis* Transcription Factor (http://planttfdb.cbi.pku.edu.cn/index.php?sp=Ath) and the alignment results of orthologous in the NCBI. Subsequently, the differentially expressed TFs were picked out from the DEGs in the IME vs. ME comparison, and between the stages in IMEs and MEs.

### Unigene quantification by real-time PCR.

A total of 32 unigenes were selected for expression profile validation by qRT-PCR. Reverse transcription (RT) reactions were performed in 3 independent biological replicates with RNA that was individually extracted from 3 independent biological samples of six types of callus, performed using a PrimeScript® RT reagent Kit with gDNA Eraser (Perfect Real Time; Takara, China). The first genomic DNA elimination reaction was conducted in a final volume of 10 μL including 2.0 μL of 5× gDNA Eraser Buffer, 1.0 μL of gDNA Eraser, 2.0 μL of total RNA, and 5 μL of RNase Free dH2O. Reactions were incubated at 42 °C for 2 min, and then maintained at 4 °C. The RT reactions (10.0 μL) were then used for the SYBR® Green qPCR assay in a 20-μL reaction mixture that included 4.0 μL of 5 × PrimeScript® Buffer 2 (for real time), 1.0 μL of PrimeScript® RT Enzyme Mix I, 1.0 μL of RT Primer Mix, and 4.0 μL of RNase Free dH2O. The reactions were incubated at 37 °C for 15 min, followed by 85 °C for 5 s, after which they were maintained at 4 °C.

Real-time PCR was performed on a Bio-Rad CFX96TM Real-Time System with SYBR® Premix Ex Taq II™ (Takara, China). Each reaction included 2 μL of product from the diluted RT reactions (cDNA solution), 1.0 μL of each primer (Additional file 1), 12.5 μL of SYBR® Premix Ex TaqTM II (2×), and 8.5 μL of RNase free water. All qRT-PCR reactions were incubated in a 96-well plate at 95 °C for 30 s, followed by 40 cycles at 95 °C for 5 s, 60 °C for 30 s, and 72 °C for 30 s. The actin gene (GenBank: AB181991) was used as the endogenous control. All reactions were run in triplicate. The specificity of each primer pair was verified by agarose gel electrophoresis and melting curve analysis. The relative expression levels of genes were calculated using the 2^-ΔΔCt^ method [49]. The gene sample in the ME library along with the CT value was selected as the calibrator, in which the expression level was set as 1.0. The relative expression levels of the same genes in the corresponding IME library were then normalized by comparison.

## Results

### Illumina sequencing and de novo assembly

With regards to the transcriptional analysis, the calli from IMEs and MEs cultured in vitro at 3d, 6d and 15d were selected, resulting in a total of 6 cDNA libraries (IME3, IME6, IME15 and ME3, ME6, ME15) that were constructed and sequenced using the Illumina HiSeq™ 2000 platform. Following data cleaning and quality checking, 155,192,839 reads (31.34 G) with Q30 values ≥80.00% were obtained from the six libraries. Among the clean reads, 100% had quality scores at Q20 level (Table 1).

**Table 1:** Output statistics of the total reads from 6 libraries

Library	Total reads (*n*)	Total nucleotides (bp)	Q20 percentage	GC percentage	Q30 percentage
IME3	28,775,750	5,808,494,474	100.00%	56.35%	80.18%
IME6	24,767,646	5,002,204,197	100.00%	56.47%	80.13%
IME15	25,514,989	5,153,360,463	100.00%	55.88%	80.00%
ME3	25,841,219	5,219,396,740	100.00%	56.10%	80.05%
ME6	25,745,846	5,200,136,819	100.00%	55.88%	80.20%
ME15	24,547,389	4,957,729,251	100.00%	55.73%	80.22%

ME3, ME6, and ME15 are mature embryos at 3 d, 6 d, and 15 d of culture, respectively

IME3, IME6, and IME15 are immature embryos at 3 d, 6 d, and 15d of culture, respectively

The high-quality reads were then merged and de novo assembled using the Trinity platform software, resulting in the generation of 331,201 transcripts with an average length of 1009.69 bp and a N50 length of 1639 bp. The total length of all transcripts was approximately 334 Mb. These transcripts were further subjected to cluster and assembly analyses, resulting in 142,221 unigenes with an average length of 657 bp and an N50 value of 1001 bp, of which 23,875 unigenes were more than 1kbin length (Table 2, Additional file 2). Open Reading Frames (ORFs) were analyzed by Getorf from the EMBOSS package and 141,393 unigenes (99.42%) had ORFs with a start codon (Table 3).

**Table 2:** Overview of the de novo sequence assembly for wheat callus

Length range	Contigs	Transcripts	Unigenes
<300	9,122,919 (98.65%)	67,091 (20.26%)	49,059 (34.49%)
300–500	60,061 (0.65%)	69,816 (21.08%)	42,416 (29.82%)
500–1000	38,359 (0.41%)	75,986 (22.94%)	26,871 (18.89%)
1000–2000	18,524 (0.20%)	74,109 (22.38%)	15,378 (10.81%)
> 2000	7933 (0.09%)	44,199 (13.35%)	8497 (5.97%)
Total number	9,247,796	331,201	142,221
Total length	554,005,887	334,411,704	93,394,731
N50 length	49	1639	1001
Mean length	59.91	1009.69	656.69

**Table 3:** ORF length of the wheat unigene

Length range	Unigene ORF
0–300	104,094 (73.62%)
300–500	16,493 (11.66%)
500–1000	11,060 (7.82%)
1000–2000	7326 (5.18%)
2000+	2420 (1.71%)
Total number	141,393
Total length	47,224,473
N50 length	558
Mean length	333.99

### Functional annotation of unigenes

**Fig. 1:**
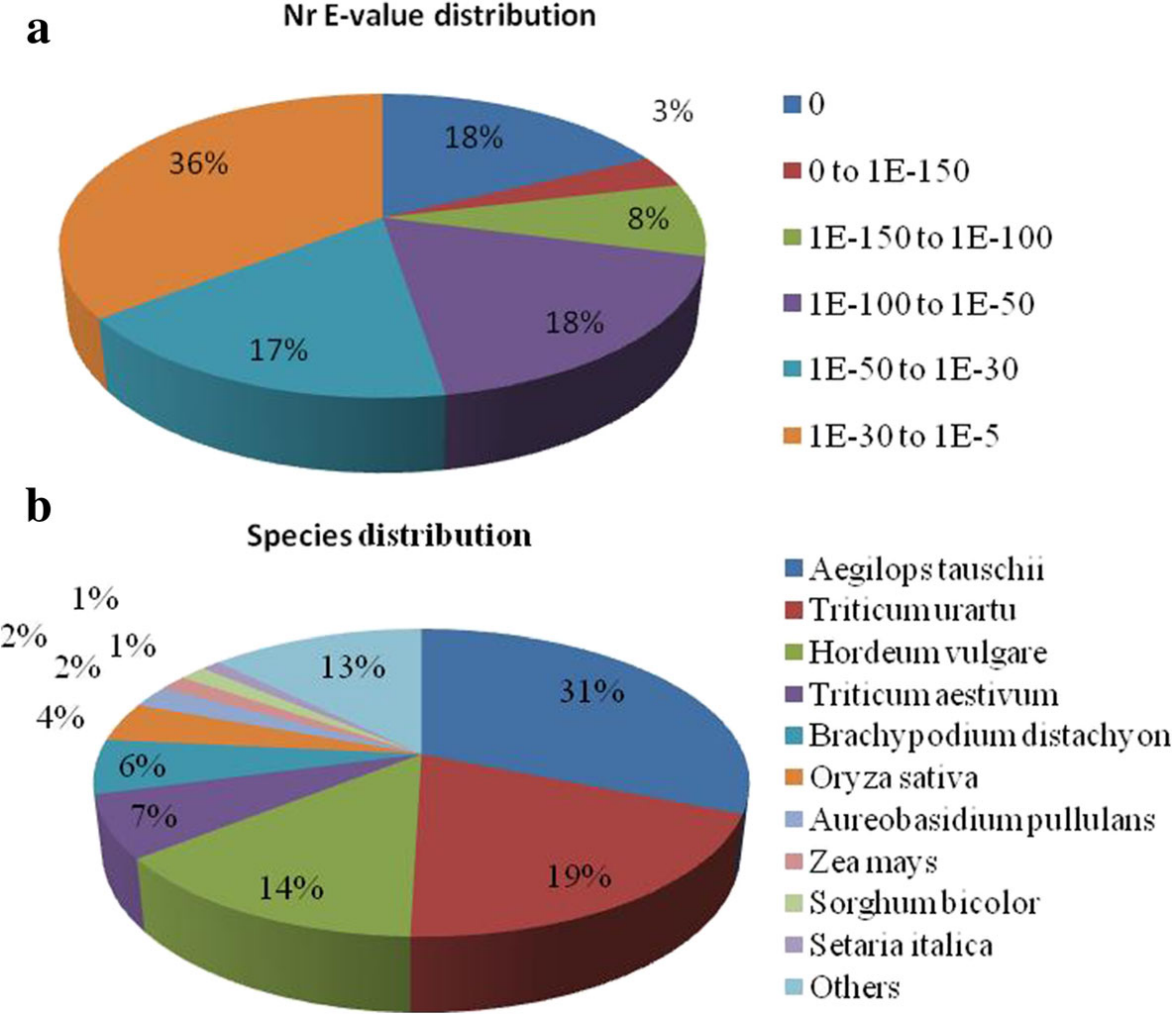
Characteristics of the homology search of unigenes against the Nr database. **a**. E-value distribution; **b**. Best hit species distribution. The cut-off values for the BLAST search were set at 1.0e^−10^

The unigenes were annotated by means of a Blastx search against the public databases. Among all 142,221 unigenes, 59,976 (42.17%) were successfully annotated by at least one database in 7 databases, i.e., Nr, Pfam, Swissprot, KOG, KEGG, GO and COG (Table 4). Furthermore, 26,529 (18.65%), 29,587 (20.08%) and 14,727 (10.36%) unigenes were annotated in the Swiss-Prot database, Pfam, and KEGG database, respectively (Additional file 3). In addition, 58,723 unigenes (41.29%) had significant matches in the NCBI non-redundant protein (Nr) database with an E-value cut-off of 10^−5^ (Table 2, Fig. 1), and 27,832 (47%) of them had an E-value lower than 1.0 e-50 (Fig. 1a). With respect to species, the top matched plant species included *Aegilops tauschii* (18,301, 31%), followed by *Triticum urartu* (11,324, 19%), *Hordeum vulgare* (8091, 14%) and *Triticum aestivum* (3869, 7%; Fig. 1b).

**Table 4:** The number and distribution of unigenes annotated in the databases

Database	COG	GO	KEGG	KOG	Pfam	Swissprot	nr	All
Unigene no.	11,513	34,761	14,727	24,345	29,587	26,529	58,723	59,976
Percentage (%)	8.10	24.44	10.36	17.12	20.80	18.65	41.29	42.17

**Fig. 2:**
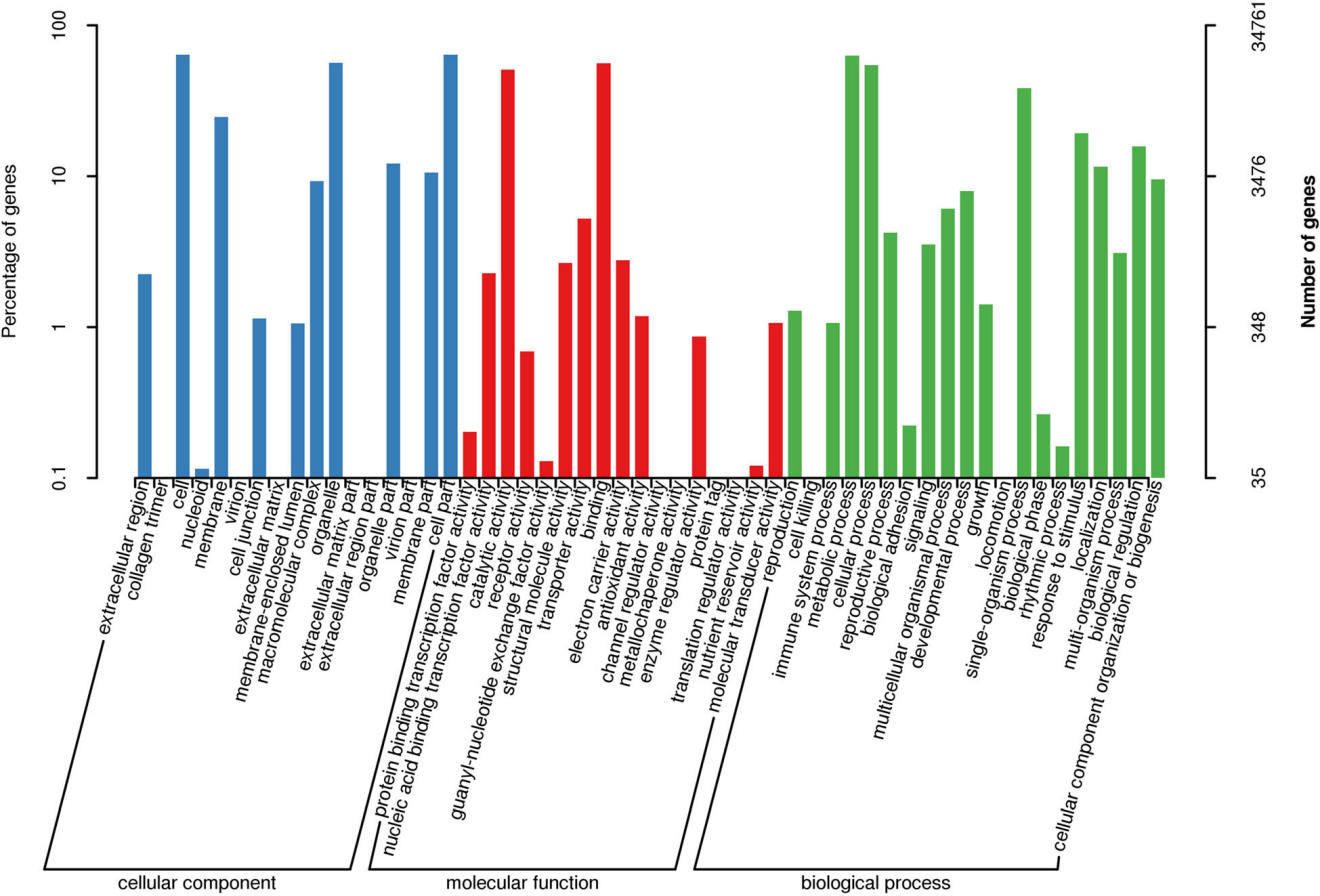
GO annotation of assembled unigenes by Blast2GO in wheat callus. A total of 34,761 unigenes were assigned to at least one GO term in 3 categories: biological process, molecular function, and cellular component. The x-axis indicates the sub-categories and the y-axis indicates the number of unigenes

To further reveal their functions, gene ontology (GO) assignments were performed using Blast2GO, with the result that 34,761 (24.44%) unigenes received at least one GO term and were classified into 3 major functional categories: cellular component, molecular function, and biological process (E-value <1E-5). For the biological process category, the majority of unigenes were annotated with “metabolic process” (21,814 unigenes), “cellular process” (18,859 unigenes) and “single-organism process” (13,313 unigenes; Fig. 1). Within the molecular function category, the highly represented GO terms included “binding” (19,497 unigenes), “catalytic activity” (17,665 unigenes) and “transporter activity” (1811 unigenes), while “cell part” (22,229 unigenes), “cell” (22,171 unigenes), and “organelle” (19,617 unigenes) were highly represented in the cellular component category (Fig. 2, Additional file 4a).

**Fig. 3:**
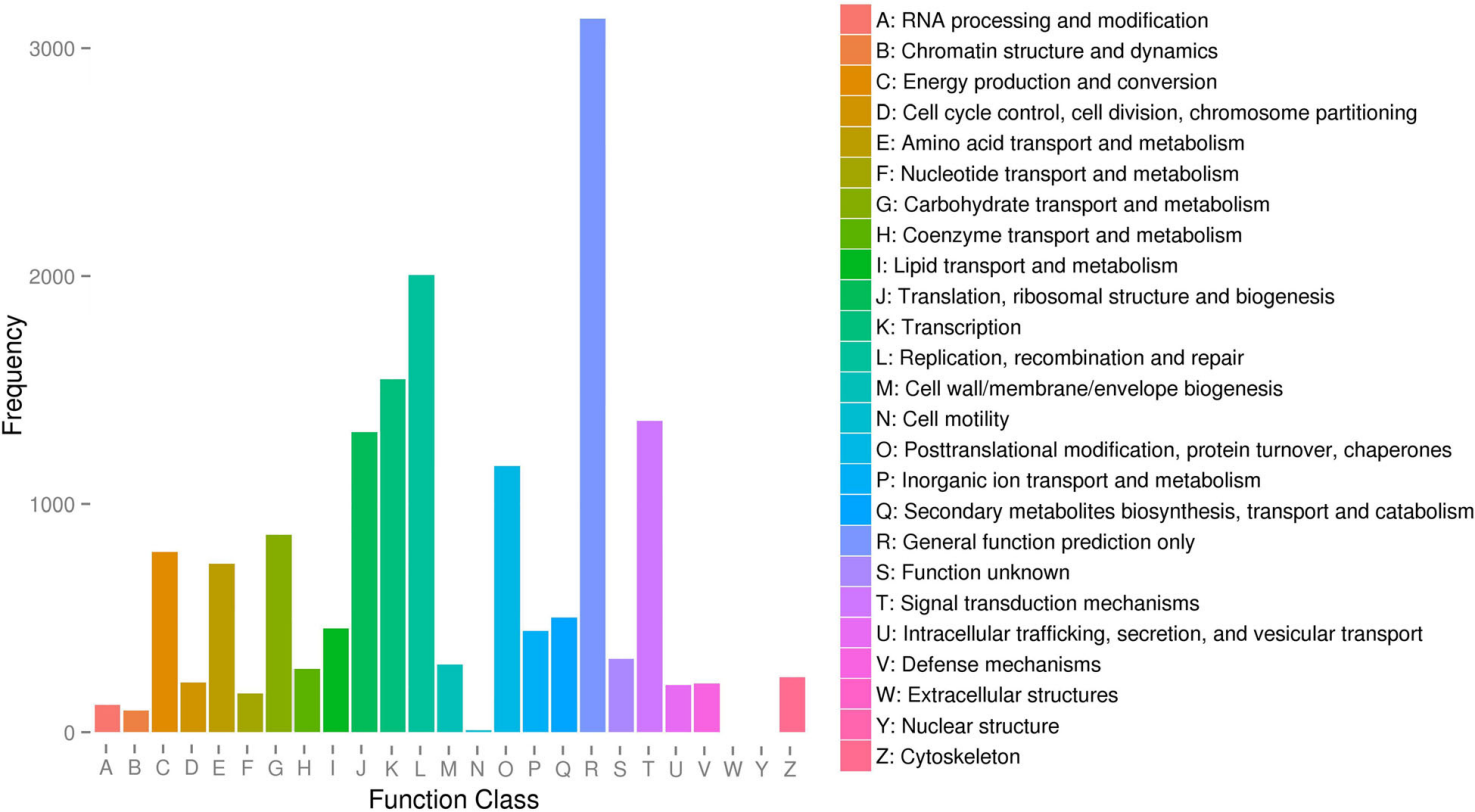
COG assignments of assembled unigenes in wheat callus. Out of 142,221 de novo assembled unigenes, 14,727 were assigned to 25 COG categories

**Fig. 4:**
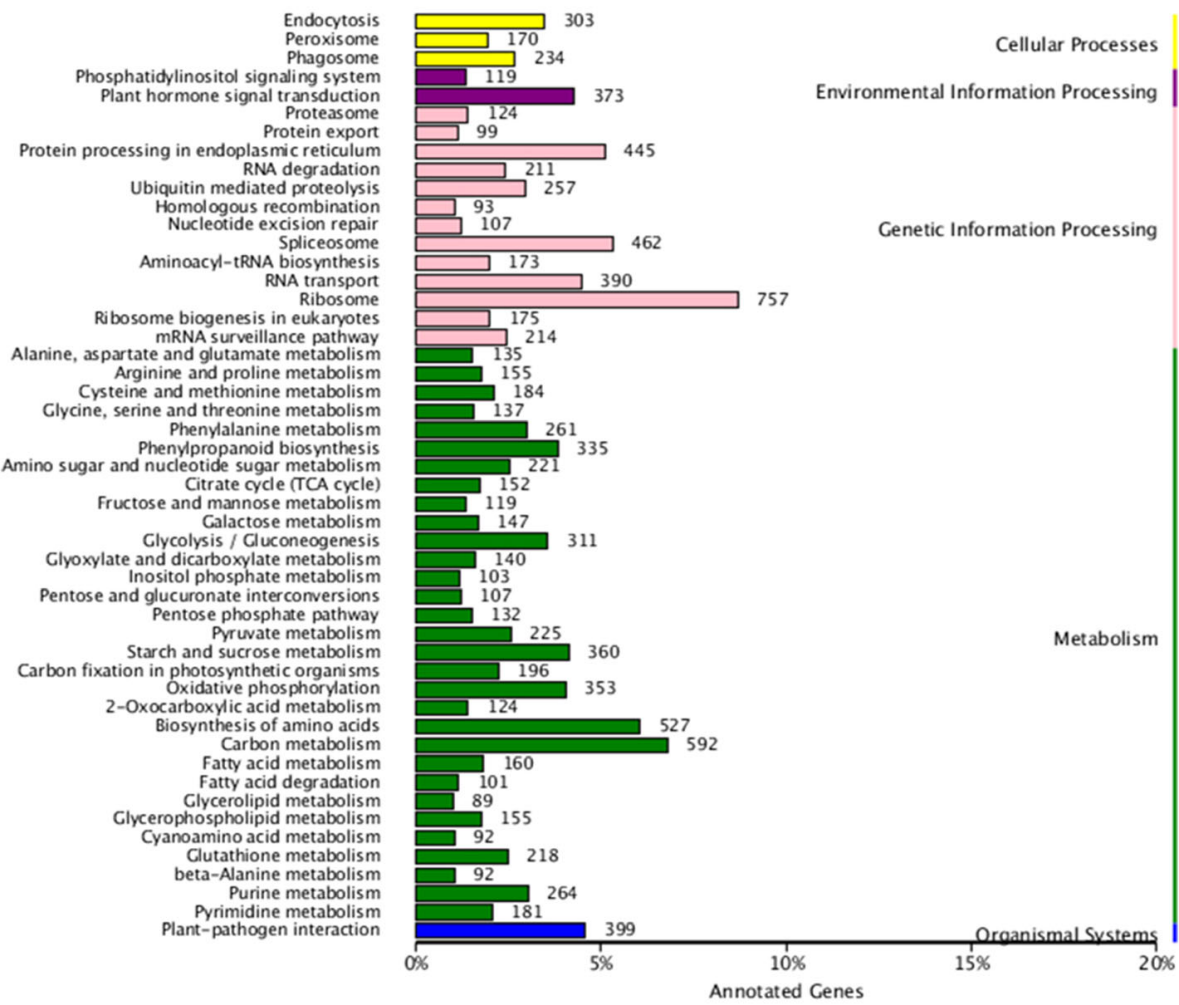
KEGG annotation of assembled unigenes in wheat callus. Distribution of the number of genes expressed in the various metabolic pathways. **a** cellular processes; (**b**) environmental information processing; (**c**) genetic information processing; (**d**) metabolism; (**e**) organismal systems

The search against the COG database resulted in 11,513 (9.79%) unigenes assigned to COG classification and divided into 25 COG categories, with “general function prediction only” (3129), “replication, recombination and repair” (2004) and “transcription” (1548) being the 3 most highly represented (Fig. 3; Additional file 4b). Metabolic pathway analysis was conducted using the KEGG orthology (KO) categories system. Consequently, 14,727 (10.36%) unigenes were assigned to 128 pathways and divided into 5 classes. In the primary pathway hierarchy, “metabolism” and “genetic information processing” were dominant (Fig. 4; Additional file 4c).

### Investigation of differentially expressed genes and functional categorization

**Fig. 5:**
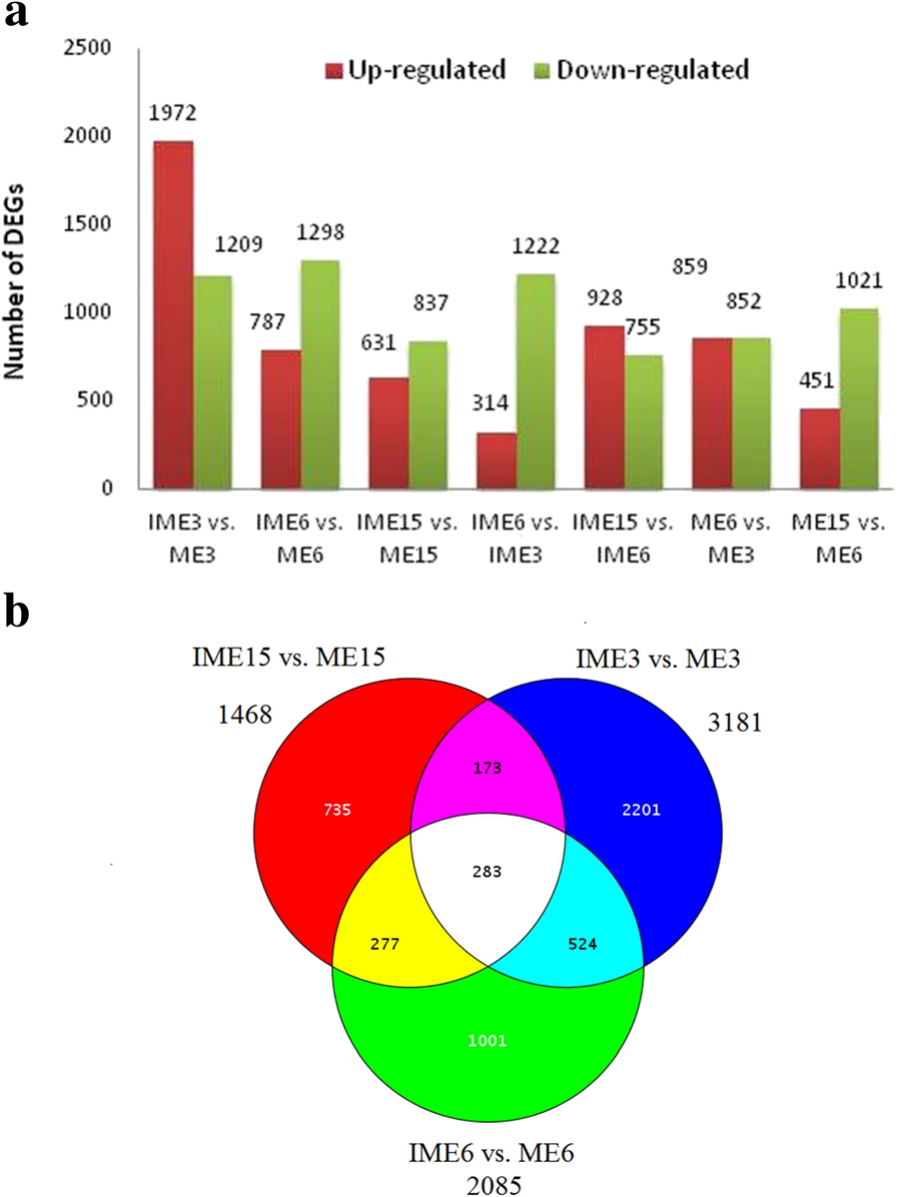
Histogram and Venn diagram of the differentially expressed genes (DEGs) in comparisons of IME vs. ME and between stages in IMEs and MEs during callus formation in wheat callus. **a** Histogram showing the number of DEGs up- or down-regulated in each comparison. **b** Venn diagrams showing similarly or distinctly regulated genes in the three comparisons of IME vs. ME

The expression levels of each unigene in the6 libraries were normalized and calculated using RPKM [47]; Additional file 5). Differentiall**y** expressed genes (DEGs) analysis in the comparisons of IME vs. ME at the 3 corresponding stages resulted in a total of 5194 DEGs in all three comparisons, with 3181, 2085 and 1468 DEGs at 3, 6 and 15 DC, respectively (Fig. 5a; Additional file 6a–c). With regards to the comparisons between developmental stages 4731 DEGs were identified (Fig. 5a; Additional file 7a-d), of which 2937 overlapped with the 5194 DEGs in the IME vs. ME comparisons (Additional file 7e).

In the IME vs. ME comparisons, 283 DEGs overlapped in all 3 comparisons, of which 266 were annotated in the 7 databases mentioned above (Fig. 5b; Additional file 6d–e). The degree of DEG overlap between the comparisons during wheat embryogenic callus formation isindicated in Fig. 5b. Functional classification indicated that 2579, 1878, and 1271 DEGs could be annotated in at least one of the 7 databases at 3, 6, and 15 DC, respectively, while 1873, 1433, and 919 DEGs could be assigned to at least one GO term at 3, 6, and 15 DC, respectively (Table 5; Additional file 8a–c).

**Table 5:** The number of differentially expressed unigenes annotated

	ALL	COG	GO	KEGG	KOG	Pfam	SWISS	Nr
IME3 vs. ME3 (no.)	2579	515	1873	713	1013	1875	1720	2545
%	81.08	16.19	58.88	22.41	31.85	58.94	54.07	80.01
IME6 vs. ME6 (no.)	1878	387	1433	542	722	1416	1265	1875
%	90.07	18.56	68.73	26.00	34.63	67.91	60.67	89.93
IME15 vs. ME15 (no.)	1271	241	919	333	431	917	831	1261
%	86.58	16.42	62.60	22.68	29.36	62.47	56.61	85.90
IME6 vs.IME3 (no.)	1302	233	916	344	489	950	830	1284
%	84.77	15.17	59.64	22.40	31.84	61.85	54.04	83.59
IME15 vs. IME6 (no.)	1450	282	1035	396	514	1037	956	1430
%	86.16	16.76	61.50	23.53	30.54	61.62	56.80	84.97
ME6 vs. ME3 (no.)	1525	422	1063	546	692	1095	956	1519
%	89.13	24.66	62.13	31.91	40.44	64.00	55.87	88.78
ME15 vs. ME6 (no.)	1274	331	897	416	526	925	789	1260
%	86.55	22.49	60.94	28.26	35.73	62.84	53.60	85.60

ME3, ME6, and ME15 are mature embryos at 3 d, 6 d, and 15 d of culture, respectively

IME3, IME6, and IME15 are immature embryos at 3 d, 6 d, and 15d of culture, respectively

**Fig. 6:**
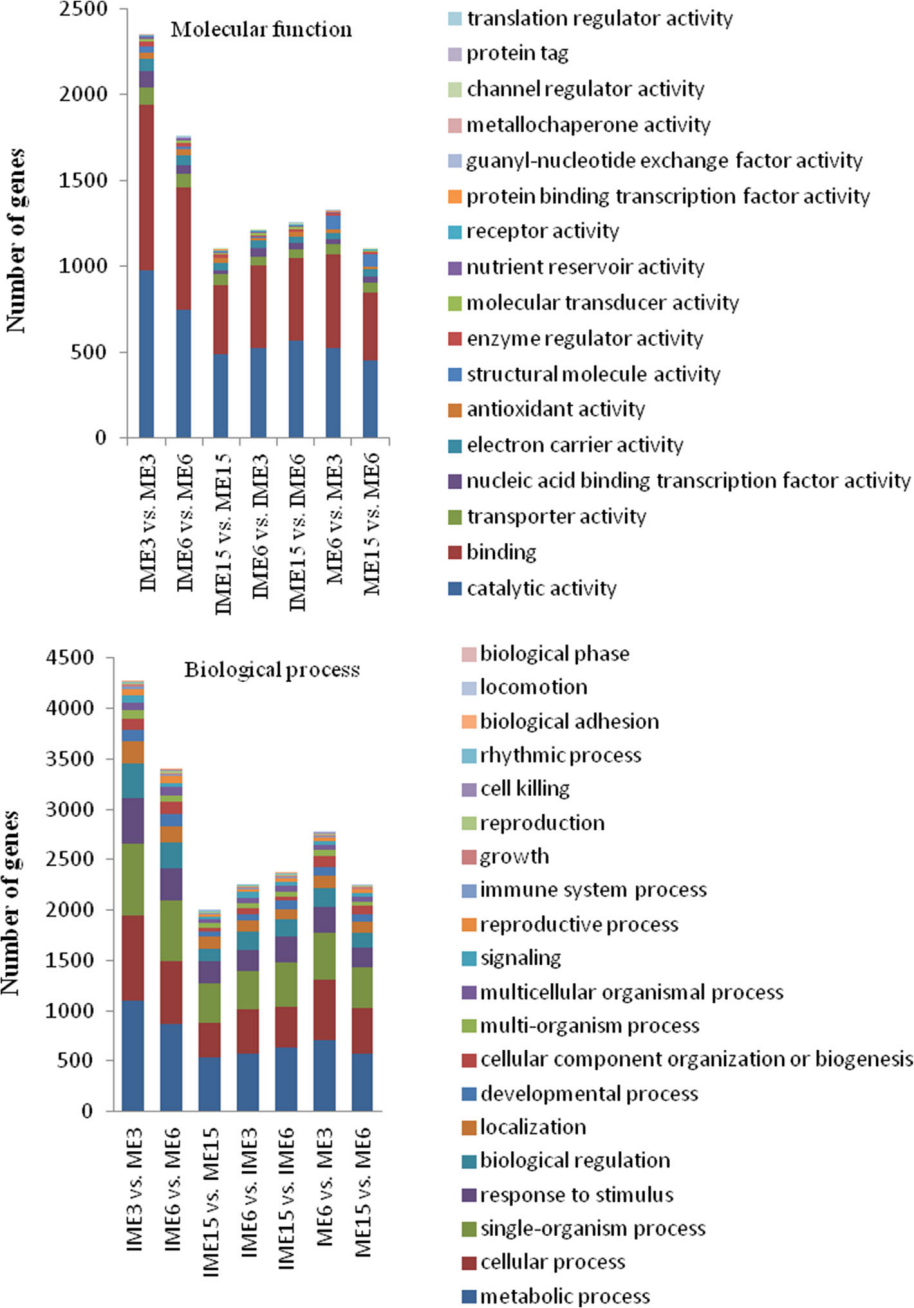
Molecular functions and biological processes of DEGs in comparisons of IME vs. ME and between stages in IMEs and MEs during callus formation based on gene ontology categories

In the comparisons between stages in IMEs and MEs, 1302, 1450, 1525 and 1274 DEGs could be annotated in at least one of the 7 databases in the IME6 vs. IME3, IME15 vs. IME6, ME6 vs. ME3, and ME15 vs. ME6 comparisons, respectively. Those assigned to GO terms included 1916, 1035, 1063, and 897 across the 4 comparisons, respectively (Table 5; Additional file 8d–g). In the biological process category of GO assignment, metabolic processes, cellular process and single-organism process were the 3 top groups in each comparison (Fig. 6a). With respect to the molecular function category, the assignments were mainly attributed to catalysis and binding (Fig. 6b).

Of the KEGG pathway annotation of DEGs in the IME vs. ME comparisons, 473 unigenes were assigned to 97 KEGG pathways in IME3 vs. ME3, while 335 unigenes were assigned to 89 KEGG pathways in IME6 vs. ME6, and 202 unigenes were assigned to 82 KEGG pathways in the IME15 vs. ME15 comparison (Additional file 9a–c).

Pathway enrichment analysis revealed that the most representative unigenes included phenylpropanoid biosynthesis, plant-pathogen interaction, starch and sucrose metabolism, and plant hormone signal transduction in the IME3 vs. ME3 comparison (Additional file 9 a). In the IME6 vs. ME6 comparison, the 4 most represented unigenes included phenylpropanoid biosynthesis, phenylalanine metabolism, starch and sucrose metabolism, and plant hormone signal transduction (Additional file 9b). In the IME15 vs. ME15 comparison, phenylpropanoid biosynthesis and phenylalanine metabolism were the prevailing categories (Additional file 9c).

In the comparisons between stages in the IMEs and MEs libraries, 228 unigenes were assigned to 79 KEGG pathways in the IME6 vs. IME3 comparison, while 253 were assigned to 86 KEGG pathways in the IME15 vs. IME6 comparison. Additionally, 393 were assigned to 93 KEGG pathways in the ME6 vs. ME3 comparison, and 326 to 84 KEGG pathways in the ME15 vs. ME6 comparison (Additional file 9d–g).

### Differently expressed TFs

**Fig. 7:**
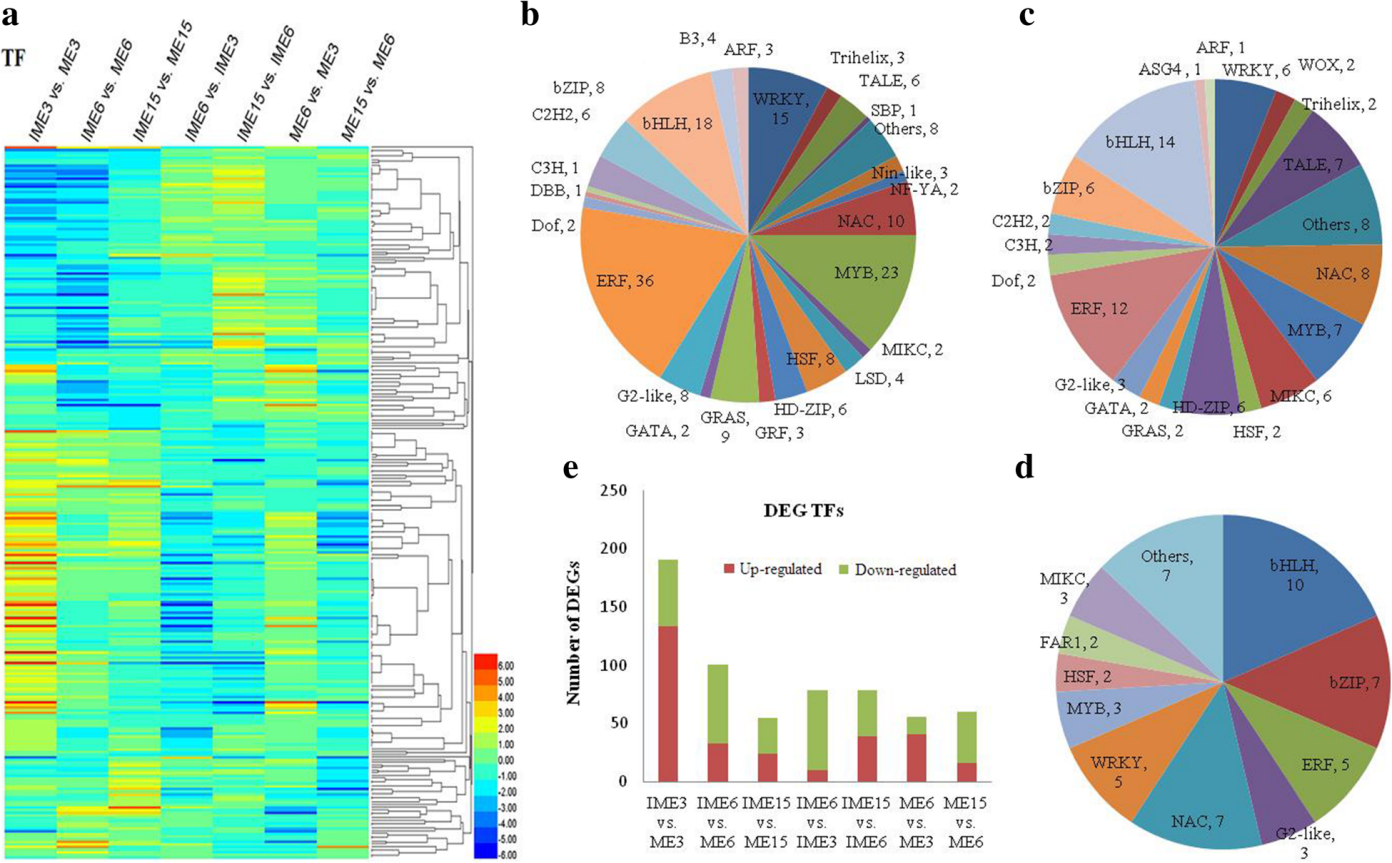
Analyses of differentially expressed TFs during wheat callus development. **a** A heat map of 271 differentially expressed TFs. Classification of TFs assigned in the three comparisons of IME3 vs. ME3 (**b**), IME6 vs. ME6 (**c**), and IME15 vs. ME15 (**d**). **e** Histogram showing the number of differentially expressed TFs up- or down-regulated in the different comparisons. IME3, IME6, and IME15 indicate calli from immature embryos at 3d, 6d, and 15d of culture, respectively. ME3, ME6, and ME15 indicate calli from mature embryos at 3d, 6d and 15d of culture, respectively

TFs possess important functions in embryogenic callus formation. All 1787 TFs were identified in this study (1748 from *Triticum aestivum* L*.*, 23 from *Arabidopsis thaliana* L*.*, and 16 from public databases; Additional file 10a). The DEGs were classified into TF families and the results indicated that there were 271 significantly differentially expressed TFs in IME vs. ME comparisons (Fig. 7a; Additional file 10b). In the IME3 vs. ME3 comparison, 133 out of the 191 TFs showed up-regulation, while 58 showed down-regulation (Fig. 7e). The prevailing family was the Apetala2 (AP2) /Ethylene Responsive Factor (ERF) family with 36 (30 up-regulated) unigenes, followed by the MYB family with 23 unigenes (16 up-regulated), the bHLH family with 18 unigenes (11 up-regulated), the WRKY family with 15 unigenes (14 up-regulated), and the NAC family with 10 unigenes (6 up-regulated; Fig. 7b).

In the IME6 vs. ME6 comparison, 101 TFs were differentially expressed with 33 being up-regulated and 68 being down-regulated (Fig. 7e). These included the bHLH family with 14 unigenes (2 up-regulated), followed by the AP2/ERF family with 12 unigenes (7 up-regulated), the NAC family with 8 unigenes (8 down-regulated), and the MYB family with 7 unigenes (1 up-regulated; Fig. 7c). In the IME15 vs. ME15 comparison in which the somatic embryo became visible in IME, there were 55 differentially expressed TFs of which 24 were up-regulatedand 31 down-regulated (Fig. 7e). The predominant TF families included bHLH with 10 unigenes (2 up-regulated), followed with 7 unigenes by NAC (1 up-regulated) and bZIP (5 up-regulated), 5 unigenes of the WRKY (5 up-regulated) and ERFs (3 up-regulated; Fig. 7d).

Comparisons between stages in the IMEs and MEs libraries provided us with 194 significantly differentially expressed TFs in total, with 87, 79, 56, and 60 in IME6 vs. IME3, IME15 vs. IME6, ME6 vs. ME3, and ME15 vs. ME6, respectively, and the up-regulated genes were 10, 39, 41, and 16, respectively (Fig. 7e). Additionally, 150 of the 271 differentially expressed TFs in IMEs vs. MEs comparisons overlapped with the 194 differentially expressed TFs in the comparisons between the stages in IMEs and MEs. Furthermore, 10 of the 271 differentially expressed TFs of IMEs vs. MEs overlapped in all 3 comparisons (Table 6). Five of the 10 TFs were selected for expression level validation by qRT-PCR and the results indicated that they were all highly associated with the RNA-Seq value (Fig. 11).

**Table 6:** Information of DEGs overlapped in IME vs. ME at three stages that are possibly involved in embryogenic callus formation and DEGs for qRT-PCR

Category	ID	Annotation	qRT-PCR	Overlapped between stages in IMEs not in MEs	Bio- process
Bio-processes-involved genes (BPGs)	comp120922_c0	Indole-3-glycerol phosphate lyase, chloroplastic; indole synthase			response to stimulus;in multi-organism process
	comp129240_c0	Cellulose synthase			
	comp129268_c1	Mobile element transfer protein; POT family			
	comp128278_c0	Cellulose synthase			reproductive process
	comp127468_c0	S-locus glycoprotein family	yes		in multi-organism process; reproductive process
	comp115979_c0	beta-expansin TaEXPB2 [*Triticum aestivum*]			in multi-organism process; reproductive process; reproduction
	comp122548_c0	Expansin-B6 [*Triticum urartu*]			
	comp124996_c0	Expansin EXPB8 [Triticum aestivum]			
	comp126585_c2	Expansin-B18			
	comp131969_c0	Expansin EXPB9 [Triticum aestivum]			
	comp123173_c0	Aldose reductase [Triticum urartu]			response to stimulus; reproductive process
	comp133735_c0	Multicopper oxidase;; Laccase-19		yes	
	comp139514_c1	RNA recognition motif; Glycine-rich RNA-binding protein			
	comp117215_c0	Salt tolerant protein [Triticum aestivum]			response to stimulus
	comp118386_c0	hypothetical protein F775_08244 [*Aegilops tauschii*]			
	comp122412_c0	Pathogenesis-related protein / dehydrase and lipid transport			
	comp123242_c0	predicted protein [*Hordeum vulgare subsp. vulgare*]			
	comp124683_c0	NmrA-like family; NADH(P)-binding; Isoflavone reductase-like protein			
	comp125897_c0	GDSL/SGNH-like Acyl-Esterase family; Protein ESKIMO 1			
	comp127496_c0	Glutaredoxin; Monothiol glutaredoxin-S5 (Rice)			
	comp129379_c0	AnnexinD4 [Aegilops tauschii]			
	comp129891_c0	Pirin-like protein			
	comp130209_c0	Cytochrome P450			
	comp131119_c0	DREPP plasma membrane polypeptide; Salt stress root protein	Yes		
	comp131357_c0	short chain dehydrogenase;11-beta-hydroxysteroid dehydrogenase 1B			
	comp132259_c0	Glycosyl transferases group 1; Sucrose synthase 1			
	comp132259_c1	Sucrose synthase 2 [Triticum urartu]			
	comp132409_c0	Glycosyl hydrolases family 17			
	comp132805_c0	Glycogenin-2 [Triticum urartu]			
	comp134609_c0	ATPase family associated with various cellular activities			
	comp135852_c0	Multicopper oxidase;; Putative laccase-5 (Rice)			
	comp137996_c3	Fructan 6-exohydrolase [Triticum aestivum]			
	comp138064_c0	Ethylene insensitive 3			
	comp138118_c0	Senescence-associated protein			
	comp140305_c0	Universal stress protein family			
	comp140523_c1	Glycosyl hydrolases family 18; Xylanase inhibitor protein 1			
	comp141536_c0	Aconitase family (aconitate hydratase)	Yes	Yes	
	comp141781_c0	hypothetical protein TRIUR3_11299 [Triticum urartu]			
	comp142326_c1	RRM in Demeter;Transcriptional activator DEMETER	Yes		
Unknown genes that overlapped between stages in IMEs not in MEs	comp120934_c0	Xyloglucan endotransglucosylase/hydrolase (XTH) protein 22	Yes	Yes	
	comp126349_c0	Xyloglucanendo-transglycosylase (XET) C-terminus	Yes	Yes	
	comp128901_c0	Root cap	Yes	Yes	
	comp128978_c0	Cortical cell-delineating protein	Yes	Yes	
	comp134128_c0	U-box domain;E3 ubiquitin-protein ligase	Yes	Yes	
	comp136525_c0	glycerol-3-phosphate acyltransferase 2	Yes	Yes	
	comp142930_c0	Reverse transcriptase Integrase core domain;gag-polypeptide of LTR copia-type	Yes	Yes	
Overlapped TFs	comp127465_c1	YABBY	Yes		
	comp131637_c0	bHLH	Yes		
	comp134166_c0	bZIP	Yes		
	comp118204_c0	MIKC			
	comp131990_c0	LSD			
	comp140577_c0	G2-like			
	comp125594_c0	ERF		Yes	
	comp126788_c0	ERF	Yes	Yes	
	comp128506_c0	ERF	Yes		
	comp129826_c0	NAC			
Overlapped SSEGs	comp111117_c0	Disease resistance protein			
	comp123801_c0	Disease resistance protein			
	comp136600_c0	Disease resistance protein			
	comp78404_c0	Disease resistance protein			
	comp131631_c0	Disease resistance protein			
	comp132305_c0	zinc finger			
	comp132823_c2	zinc finger	Yes		
	comp136499_c1	zinc finger			
	comp136627_c0	zinc finger			
	comp124435_c0	LTP			
	comp141004_c1	LTP			
	comp146809_c0	LTP	Yes		
	comp126873_c0	LEA			
	comp113034_c0	LEA			
	comp113890_c0	LEA			
	comp129476_c0	LEA	Yes		
	comp131074_c0	LEA			
	comp131947_c0	LEA			
	comp136871_c0	LEA			
	comp117547_c0	Calcium-dependent			
	comp120829_c0	Calcium-dependent			
	comp121468_c3	Calcium-dependent			
	comp122478_c2	GST			
	comp134985_c3	GST			
	comp162414_c0	Auxin-induced protein			
	comp125890_c1	Peroxidase			
	comp126327_c4	Peroxidase			
	comp128155_c1	Peroxidase			
	comp129605_c0	Peroxidase			
	comp129803_c0	Peroxidase		Yes	
	comp130824_c1	Peroxidase	Yes		
	comp130824_c2	Peroxidase			
	comp138541_c1	Peroxidase			
	comp140208_c0	HSP			
	comp124012_c0	Germin-like protein	Yes		
DEGs not overlapped that for qRT-PCR	comp116720_c1	Heat shock 70 kDa protein	Yes		
	comp125045_c0	Oleosin 16 kDa	Yes		
	comp125097_c0	Zinc finger CCCH domain-containing protein	Yes		
	comp125797_c0	Auxin-induced protein 1	Yes		
	comp127206_c0	WAT1-related protein	Yes		
	comp128173_c1	GDSL esterase/lipase At2g40250 (Precursor)	Yes		
	comp128869_c0	B3 domain-containing protein	Yes		
	comp131743_c0	–	Yes		
	comp133896_c0	Desiccation-related protein	Yes		
	comp134203_c0	Cytochrome P450 71C3	Yes		
	comp137615_c0	Antimicrobial peptide 2d (Precursor)	Yes		

### Somatic embryogenesis-related genes in wheat callus

**Fig. 8:**
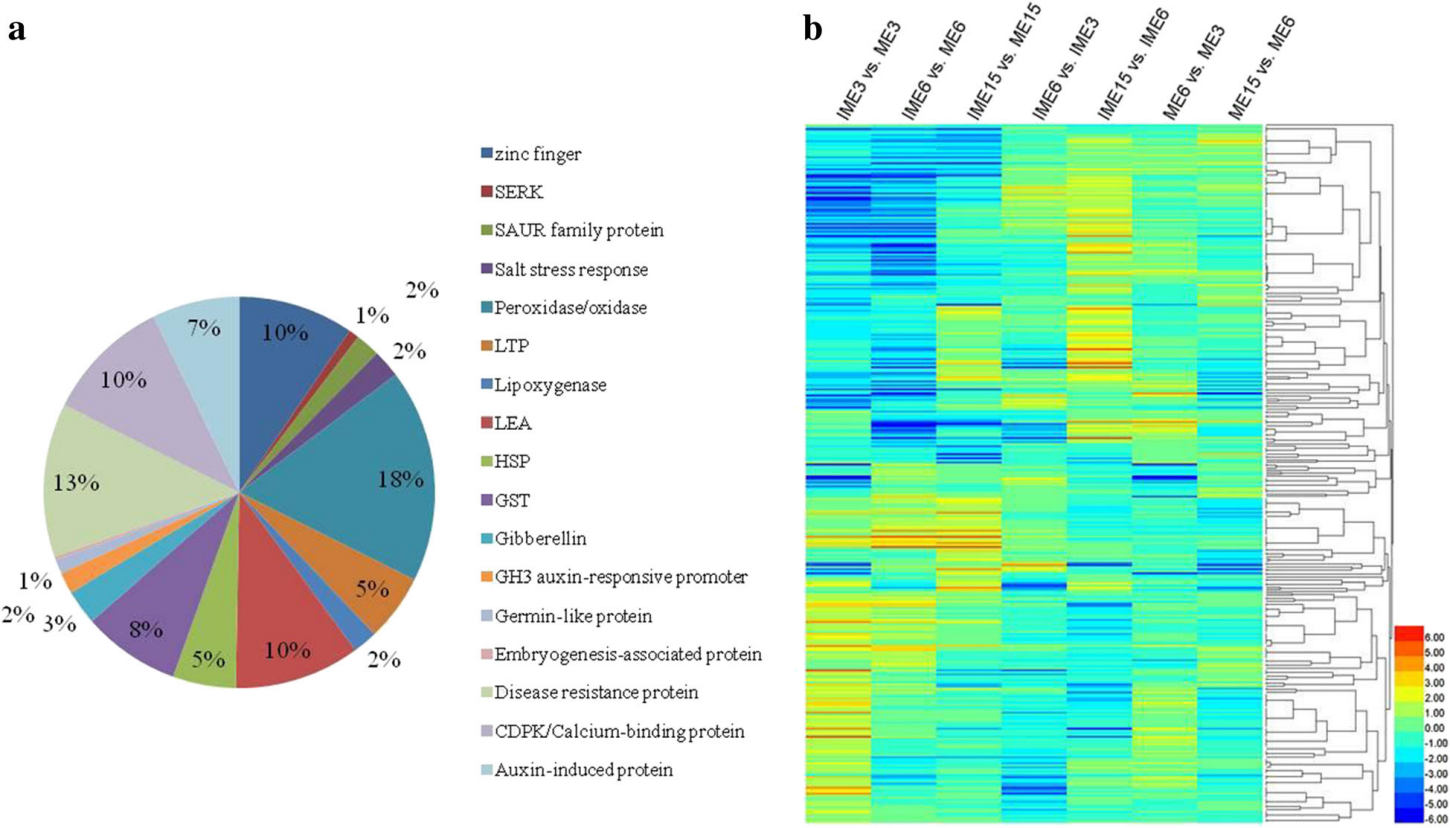
Analyses of differentially expressed somatic embryogenesis related genes during wheat callus development. **a** Expression of 346 differentially expressed somatic embryogenesis related genes. **b** The distribution of 346 somatic embryogenesis related genes in comparisons of IME vs. ME at the 3 stages. Numbers represent the percentage of genes out of the differentially expressed genes in each comparison

With respect to embryogenic callus formation, except for TFs, there were 346 DEGs identified in at least one of IME vs. ME comparisons including stress related genes or homologues to the previously annotated genes that were potentially somatic embryogenesis related genes, such as calcium-dependent protein kinase (CDPK)/calmodulin (CAM) [50, 51], germin-like protein (GLP) [52, 53], glutathione s-transferase (GST) [54], late embryogenesis abundant (LEA) proteins [55, 56], zinc finger [57, 58], non-specific lipid-transfer protein (LTP) [59], heat-shock protein (HSP) [60], indole acetic acid induced protein (Aux/IAA), and other aux related genes [4] (Fig. 8a). Of the 346 stress and somatic embryogenesis related genes (SSEGs), 35 overlapped in the 3 comparisons of IME vs. ME (Table 6). Five of the 35 SSEGs were selected for expression level validation by qRT-PCR and the results indicated that they were all highly correlated with the RNA-Seq value (Fig. 11). Auxin genes play an important role in plant cell dedifferentiation and redifferentiation [4, 61]. In this study, 44 DEGs (38 from the 346 SSEGs and 6 from the 271 TFs) were related to auxin, i.e., auxin-responsive protein (10), auxin efflux carrier component (2), auxin-induced protein (9), auxin-repressed protein (2), auxin transporter-like protein (2), auxin response factor (ARF, 4), small auxin-up RNAs (SAURs) family protein (7), GH3 auxin-responsive promoter (6), indole-3-acetaldehyde oxidase (1), and other auxin-related proteins were identified in IME vs. ME comparisons (Additional file 11a). The expression of 346 SSEGs in the 6 libraries is shown in Fig. 8b. Interestingly, 217 of them overlapped with the comparisons between stages in IME and/or ME (Additional file 11b).

The KEGG pathways associated with plant hormone signal transduction in IME vs. ME comparisons, with regards to auxin related genes, included *GH3* and *SAURs* which were down-regulated at 15d, as well as *GH3* which was up-regulated at 3d and 6d. SAUR was mix regulated (up and down) at 3d and 6d, and AUX1 was down-regulated at 3d and up-regulated at 6d. In the comparisons between stages in IMEs, ARF was down-regulated in IME6 vs. IME3 and in IME15 vs. IME6, while GH3 was down-regulated in the IME15 vs. IME6 comparison, and most of the auxin related genes are down-regulated during embryogenic callus formation from 3d of callus initiation to stage 6d of visible embryogenic callus, and to the somatic embryo generation at 15d in IME (Additional file 12). This implies that the majority of the auxin related genes are regulated in an intricate program in embryogenic callus formation.

### Expression and GO analysis of the common DEGs in IME vs. ME comparisons

**Fig. 9:**
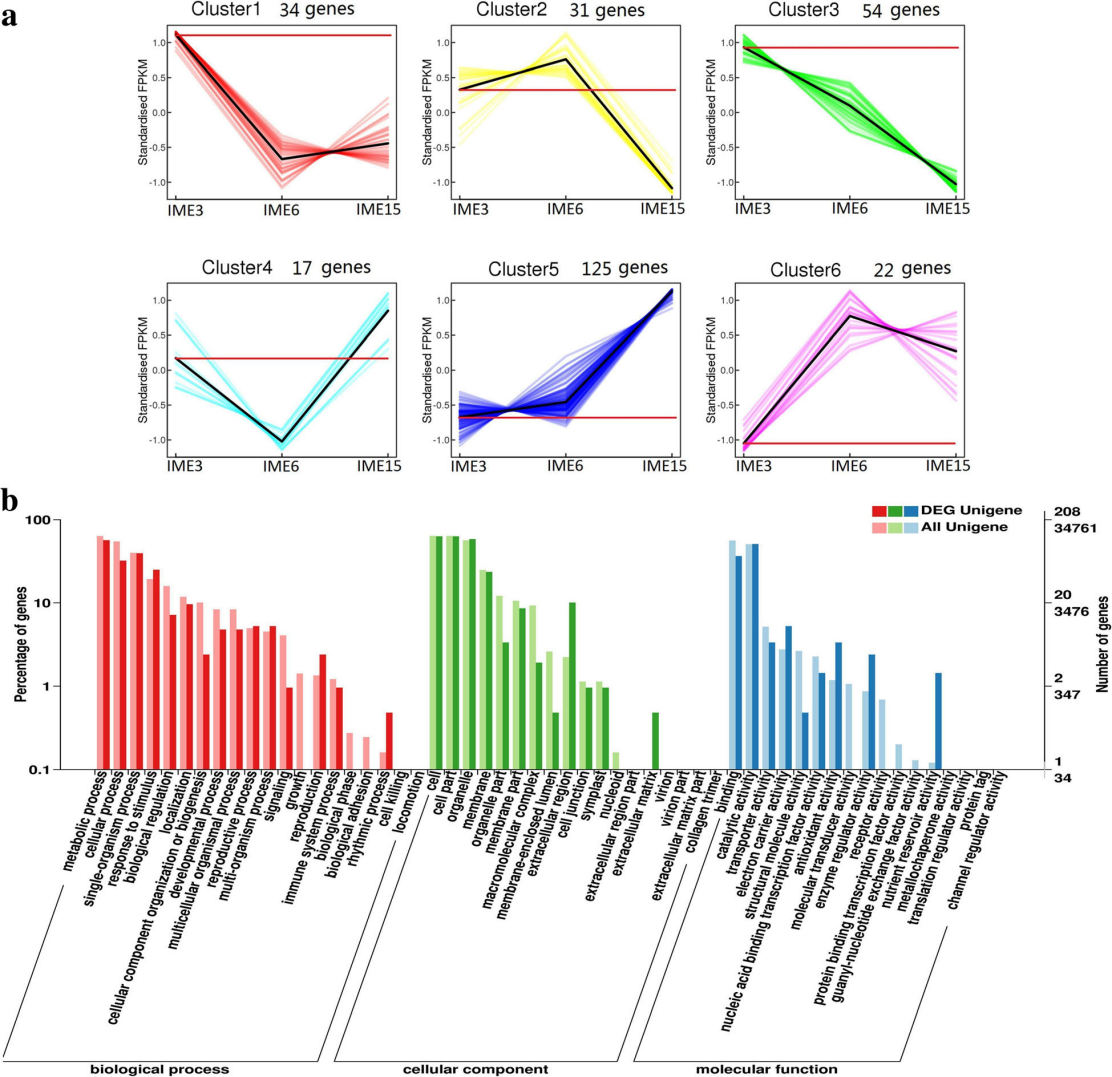
Analyses of 283 common differentially expressed genes in IME vs. ME at three stages during embryogenic callus formation in wheat. **a** Cluster analysis of 283 common DEGs in IME vs. ME at three stages based on K-means method. 283 common DEGs were divided into 6 distinct temporal expression profiles in IME. **b** GO Functional categorization of 283 common differentially expressed genes based on gene ontology

Of the 5194 DEGs in IMEs vs. MEs, 283 were simultaneously differentially expressed in all the three comparisons. Of the 283 overlapping DEGs, 35 overlapped in the above mentioned 346 SSEGs, while 10 overlapped in the above-mentioned 271 TFs. The expressions of the 283 DEGs were clustered into 6 groups in the IME libraries (Fig. 9a).

**Fig. 10:**
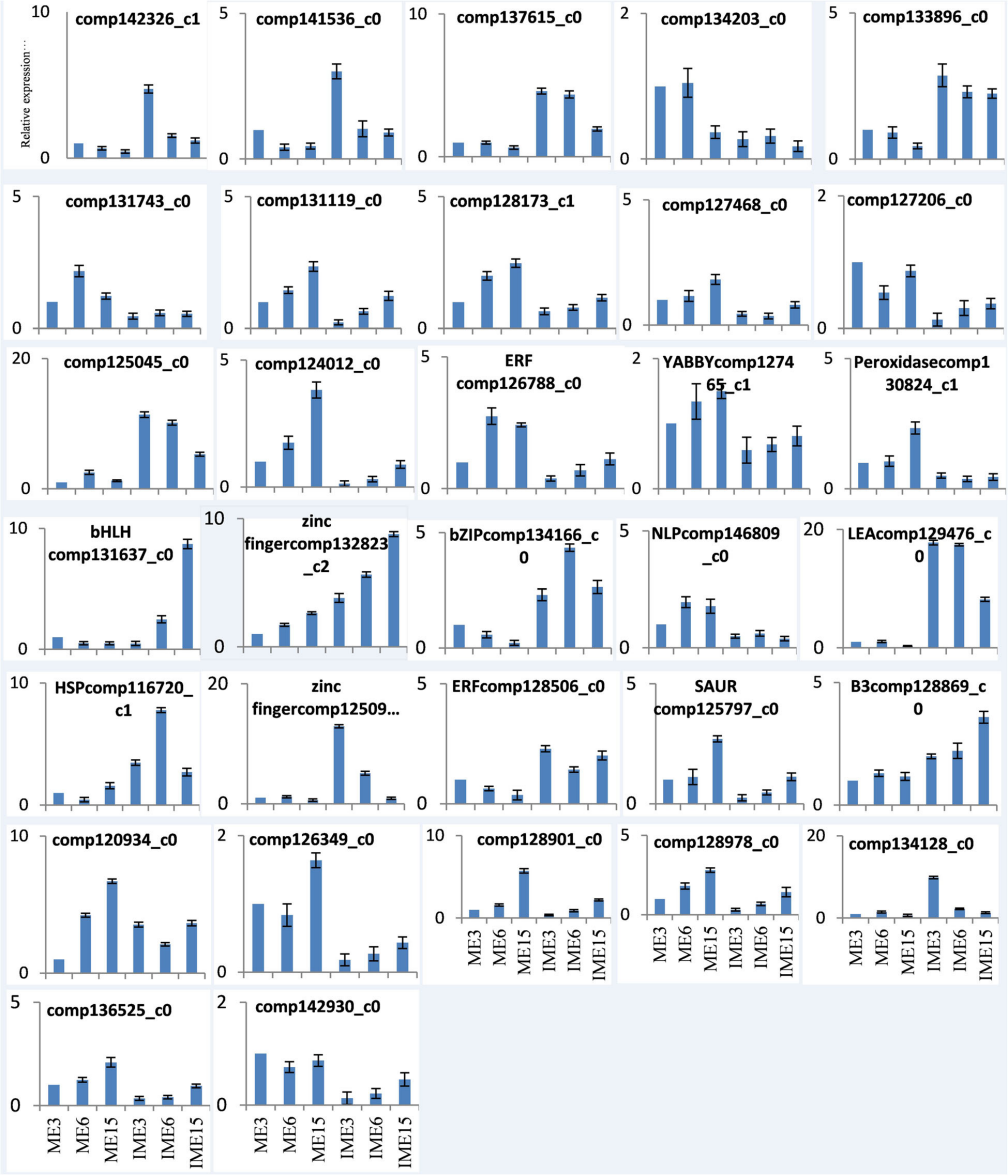
Relative mRNA levels of 32 DEGs in wheat callus were determined by quantitative RT-PCR analyses. Genes were normalized to the actin gene (GenBank: AB181991). Experiments were repeated in triplicate. Error bars represent one standard deviation (SD). Relative expression level of genes in IME were presented as fold-change (mean ± SD) compared according to ME of 1.0

**Fig. 11:**
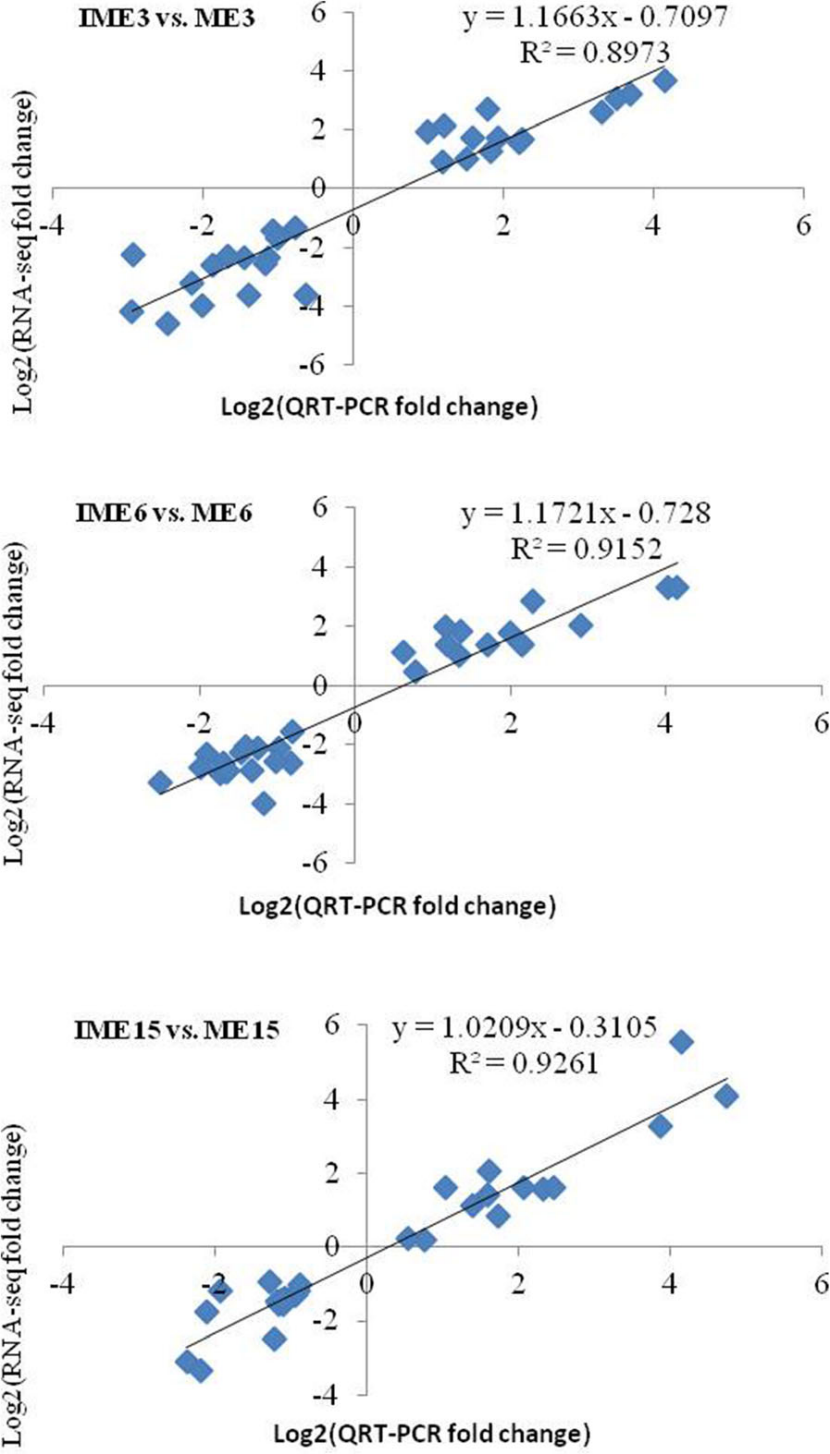
Correlation analysis of log2 fold change values obtained from RNA-Seq and qRT-PCR for 32 DEGs in the comparisons of IME vs. ME. RNA-Seq fold change refers to the ratios of RPKM values of IME to ME for selected genes, while Q-PCR fold change is the relative quantity of IME normalized to expression level of ME

GO function analysis of the 283 overlapped DEGs indicated that 208 unigenes received at least one GO term and were classified into 3 major functional categories: cellular component, molecular function, and biological process. For the biological process category, the majority of unigenes were annotated with metabolic process (99 unigenes), single-organism process (78 unigenes) and cellular process (66 unigenes; Fig. 9b). The DEGs related to processes of response to stimulus (52 unigenes), multi-organism process (11 unigenes), reproductive process (11 unigenes), and reproduction (5 unigenes) possessed a significantly higher percentage in the 283 overlapped DEGs than in all unigenes (Fig. 9b). The 52 unigenes related to response to stimulus process included 18 of the 35 SSEGs and 2 of the 10 TFs, suggesting that somatic embryogenic formation was intimately related to the response to stimulus process. Therefore, the remaining 32 unigenes may also be involved in somatic embryogenic formation. With regards to multi-organism processes, reproductive processes and reproduction in biological processes, 11, 11 and 5 unigenes were further merged into 16 unigenes due to the involvement of some genes in multiple functions. Three of the merged 16 unigenes belonged to 35 SSEGs and the 10 TFs, and the remaining 13 genes may also be involved in somatic embryogenic formation. Of the 13 unigenes, 5 (comp115979_c0, comp122548_c0, comp124996_c0, comp126585_c2, comp131969_c0) were simultaneously involved in all 3 processes. Furthermore, the 32 and 13 unigenes possibly involved in somatic embryogenic formation were merged into 39 unigenes by the bio-process terms of the GO analysis (Table 6). Four of the 39 bio-processes-involved unigenes (BPGs) were selected to perform expression level analysis by qRT-PCR (Fig. 10). The results indicated that their expression levels were highly associated with the RNA-Seq value (Fig. 11).

Of all 283 overlapping DEGs in all 3 comparisons of IME vs. ME, 12 containing 5 annotated genes (1 from the 35 SSEGs, 2 from the 10 TFs, and 2 from the 39 BPGs mentioned above) and 7 unknown genes (Table 7) showed significant differences in the different stages of IMEs, but did not show any significant difference in the different stages of MEs, indicating that the 7 unknown genes might be related to embryogenic callus formation. The qRT-PCR results validated that the 7 unknown genes and one (comp126788_c0) of the 5 annotated genes showed highly consistent expression levels with RNA-Seq (Fig. 10). Therefore, expression comparative analysis and function analysis showed that the above-mentioned 10 IFs, 35 SSEGs, 39 BPGs and 7 unknown DEGs might contribute to embryogenic callus formation.

## Discussion

Bread wheat is one of the major human food crops and has been subject to the application of biotechnology for crop improvement. High plant regeneration efficiency forms the basis for biotechnology by genetic transformation. Numerous studies have aimed to improve regeneration efficiency in wheat by optimizing the culture conditions, the explant physiological traits, and the genotypes. Unfortunately, the regeneration efficiency remains poor. Since embryogenic calli can be harnessed to generate plants with improved tissue culture efficiency, illustration of the molecular mechanism of embryogenic callus formation is helpful for manipulation of the regeneration process.

The expression of the somatic embryogenesis-related genes and signaling regulation has been researched in plants by comparative analysis between the somatic embryos and zygotic embryos [4, 42, 62] or during somatic embryogenesis [43] using NGS. In the present study, we obtained the transcriptome of the embryogenic calli derived from IMEs and the non-embryogenic calli derived from MEs following culturing for 3d, 6d, and 15d. The de novo assembly generated 142,221 unigenes and the comparisons of IME vs. ME at 3 stages provided us with 5194 DEGs; more DEGs were identified in the comparisons at 3 DC and 6 DC than at 15 DC. Additionally, 4731 DEGs were identified in the comparisons between the stages in IMEs and MEs, suggesting that the regulatory pathway exists in embryogenic callus formation in bread wheat.

Previous studies have identified genes that are potentially involved in somatic embryogenesis, including some genes that are related to stress response [4–6, 43] and auxin processes [40]. Several studies have revealed that stress plays a key role in somatic embryogenesis [63]. In the 3 comparisons of IME vs. ME, 346 SSEGs of DEGs were identified including HSP, GLPs, LEA, CDPK/CAM, GST, and LTP, which are potentially involved in embryogenic callus formation. Additionally, 35 of them overlapped in all 3 comparisons of IME vs. ME.

LTP is related to embryogenic callus development through the regulation of the embryogenic callus formation pathway [59], and Ca^2+^ has been suggested to play an intermediary role during plant embryogenesis [50, 51]. GST was previously shown to be induced by various stresses involved in the SE through complex interactions with other transcriptional regulators and auxin metabolism [54, 64]. Germins and GLPs are thought to play a significant role in stress and in somatic embryogenesis in wheat and Germins had oxalate oxidase activity [52, 53], 6 differently expressed genes related to GLPs (4) and oxalate oxidase (2) were identified in IME vs. ME comparisons (Additional file 11 b-c), while LEA, ABA and ethylene are stress-related substances for the acquisition of embryogenic competence by plant cells [55, 56]. Many genes thatrespond to various abiotic stresses have previously been found to be induced by auxin [4]. For example, *Hsps* in carrot somatic cells was found to be an auxin-responsive gene during somatic embryo development [60].

Auxin as a plant growth hormoneis considered to be essential for the initiation of cell division and differentiation [65] before it can express embryogenic competence [66], as well as in the initiation of embryogenesis from somatic tissues [67]. Auxin accumulation was previously detected in developing somatic embryos in *Arabidopsis* and carrot [68, 69], and the endogenous auxin concentration reached a peak at the embryogenic callus stage during somatic embryogenesis [40]. In sunflower, an endogenous auxin pulse was believed to be involved in the induction of somatic embryogenesis [70]. Exogenous auxin application and endogenous auxin content were both determining factors during the induction phase. The level of free and conjugated IAA was previously found to be highly regulated with embryonic callus formation in wheat [26]. In this study, KEGG analysis of DEGs in IME vs. ME comparison demonstrated that many genes were involved in plant hormone signal transduction with down-regulation during the process of embryogenic callus formation (Fig. 8a). Research of SE identification and characterization indicated that stress-related genes and proteins were associated with SE in stress-induced acquisition [71]. Thus the 346 DEGs of SSEGs, especially the 35 overlapping DEGs, might be involved in embryogenic callus formation.

TFs are important factors involved in plant development as well as in regeneration process. A series of studies on SE development revealed that complex transcription regulation networks existed in cell differentiation, maintaining embryogenic competency, embryogenic patterning, meristem maintenance, as well as roles in stress and hormone-mediated signaling [40, 44]. In this study, we identified 271 differentially expressed TFs in the comparisons of IME vs. ME, 10 of which were simultaneously differentially expressed in all 3 comparisons. Of the 271 TFs, bHLH, AP2/ERF, b-ZIP, and WRKY were highly represented.

Zinc finger family proteins have been proven to be involved in stress, the regulation of plant cell death and callus differentiation [57, 58]. BohLOL1, encoding an LSD1-like zinc finger protein in bamboo and participating in growth regulation and response to biotic stress, was up-regulated by auxins, cytokinins, and hydrogen peroxide in in vitro culture [72]. PEI1, encoding a protein containing a Cys3-His zinc finger domain, is an embryo-specific transcription factor that plays an important role during *Arabidopsis* embryogenesis [73]. The over expression of OsLSD1, a rice zinc finger protein, accelerated callus differentiation in transformed rice tissues [58]. In the present study, the up-regulated unigenes that encode the zinc finger family protein in the IME vs. ME comparisons were 15/18, 8/18, and 0/3 at 3, 6, and 15 DC, respectively (Additional file 8). The ratio of the up-regulated DEGs was higher at the phase of embryogenic callus initiation than at the development and completed phase, indicating that they have a regulatory role in wheat embryogenic callus formation.

A member of the AP2/ERF domain family BABY BOOM (BBM) was identified as a marker of SE in cell cultures of *Brassica napus* (*B. napus* L.)*.*The ectopic expression of BBM can lead to somatic embryogenesis in *Arabidopsis* and *B. napus* [21, 74], while over-expression can induce embryo formation and enhance regeneration ability in tobacco [75], sweet pepper [76], and in cacao [77]. The *A. thaliana* EMBRYOMAKER (EMK), another member of the AP2/ERF domain family, plays a redundant role in the maintenance of embryonic cell identity. Ectopic expression of AtEMK can promote the initiation of somatic embryos from cotyledons [78, 79] revealed that AP2/ERF expression was related to the dramatic regulation of somatic embryogenesis in *Hevea*. In the present study, there were 20 AP2/ERF genes differentially expressed in the comparisons of IME vs. ME at 3 stages (Additional file 8), with 2 genes (comp126788_c0, comp125594_c0) being differentially expressed in IME vs. ME and in the comparisons between stages in IME, but not in comparison between stages in ME. Thus these AP2/ERF genes may be related to embryogenic callus formation in wheat.

Members of bHLH are involved in growth, developmental, and abiotic stress responses [80], axillary meristem formation [81]. They also participate in epidermal cell type specification in *Arabidopsis* [82], and are involved in brassinosteroid and abscisic acid signaling in rice and *Arabidopsis* [83]. BIM1, a bHLH protein controlling *Arabidopsis* embryonic patterning and interacting with the auxin and BR signaling pathways [84], which interacts with other proteins and TFs such as the key regulator MYB families that involved in stress response and regulated embryogenesis pathways [85]. In this study, the ratio of up-regulated unigenes of bHLH in IME vs. ME comparisons was 12/19, 3/18, and 2/11 at 3, 6, and 15d, respectively, suggesting that a complex regulation exists in embryogenic callus formation in wheat. Additionally, the majority of MYB genes was up-regulated in embryogenic initiation and showed a relatively low expression level during somatic embryo development, indicating that the members of these 2 families play important roles in embryogenic callus formation.

The WRKY TFs have been shown to be involved in the response to biotic and/or abiotic stresses [86], and were found to be up-regulated during SE in plants. The expression of the member genes in this family was shown to be associated with embryogenic callus formation [87]. In this study, the DEGs at 3 DC possessed the highest numbers of WRKY than other stages, with the ratio of up-regulated unigenes in IME vs. ME comparisons being 14/15, 2/6, and 5/5 at 3, 6, and 15d, respectively.

B3 domain transcription factors in *Arabidopisis* (LEC2, FUS3 and ABI3) encode regulatory proteins involved in embryogenesis and the induction of somatic embryo development [16]. LEC1 (LEAFY COTYLEDON1), a CCAAT box-binding factor, along with LEC2 and FUS3, are essential for plant embryo development. For instance, LEC2 promotes embryogenic induction in the somatic tissues of *Arabidopsis* [88]. Furthermore, cacao*TcLEC2*, a functional ortholog of *AtLEC2*, is involved in similar genetic regulatory networks during cacao somatic embryogenesis [89]. In this study, 4, 1, and 1 B3 TFs were differentially expressed in IME vs. ME at 3, 6 and 15d, with all of them being up-regulated. Three of them were differentially expressed in the comparison between stages in IME and/or ME.

The MADS-domain transcriptional regulator *AGAMOUS-LIKE15* (*AGL15*) had been reported to enhance somatic embryo development in *Arabidopsis* and soybean when ectopically expressed [90]. Soybean orthologs of the *Arabidopsis* MADS box genes, AGAMOUS-Like15 (GmAGL15) and GmAGL18, constitutively expressed increased embryogenic competence of explants. ABI3 and FUSCA3 were found to be directly up-regulated by GmAGL15 [22]. WUSCHEL controls meristem function by direct regulation of cytokinin-inducible response regulators, which was highly expressed during early somatic embryo development [91]. In the present study, MADS and wux genes were differentially expressed at 3d and 6d. Histone H3.3 participates in the epigenetic transmission of active chromatin states in animal and Histone H3 showed appreciable levels in the somatic embryos at all stages of somatic embryo development in Alfalfa [92, 93], 15 Histone DEG was identified with 3 of H3 in IME vs. ME comparisons (Additional file 11c).

Somatic embryogenesis is a complex process. TF with auxin and stress treatment were essential for the acquisition of embryogenic competence by carrot somatic cells [5]. In this study, many stress factors including oxidative, heat and salt stress, were differentially expressed in the comparisons. Thus, the Aux, TF, and stress related genes regulated the embryogenic callus formation. Except for SSEGs and TFs, there were 39 genes that were present in all 3 comparisons of IME vs. ME and were involved in GO biological process of response to stimulus, in multi-organism process, reproductive process and reproduction, function as expansin, cellulose synthase, mobile element transfer protein, multicopper oxidase, ATPase family associated with various cellular activities (AAA), glutaredoxin, ethylene insensitive 3, senescence-associated protein, universal stress protein family and pathogenesis-related protein/dehydrase and lipid transport, and were perhaps related toembryogenic callus formation.

The 7 unknown DEGs presented in three of the IME vs. ME comparisons and in the comparisons between stages in IMEs but not in MEs, function as xyloglucan endotransglucosylase/hydrolase (XTH) protein 22, xyloglucan endo-transglycosylase (XET) C-terminus, root cap, cortical cell-delineating protein, U-box domain/E3 ubiquitin-protein ligase *A. thaliana*, glycerol-3-phosphate acyltransferase 2 *A. thaliana*, gag-polypeptide of LTR copia-type/ reverse transcriptase Integrase core domain (Table 6). XTH, a cell wall-modifying enzyme, can act as cell wall-loosening enzymes [94], and may possess XET or endohydrolase activities [95], indicating their special roles in embryogenic callus formation.

## Conclusion

In summary, the combination of tissue culture of wheat embryos and RNA-seq approaches by global gene expression patterns during embryogenic callus formation were performed to investigate the regulatory genes in embryogenic callus formation. Expression and function analysis indicated that the DEGs of 271 TFs (10 overlapped in all 3 comparisons of IME vs. ME), 346 SSEGs (35 overlapped in all 3 comparisons of IME vs. ME), 39 BPGs, and 7 unknown unigenes that overlapped in IME vs. ME and in comparisons between stages in IMEs, but not in any one of comparison in ME, might play an important role in embryogenic callus formation in wheat. The present comparative profiling provided new insights into the molecular mechanisms for the regulation of embryogenic callus formation processes. However, as molecular markers are required to follow specific events during embryogenic callus formation, further experiments are required to evaluate the interaction between stress and auxin signaling during embryogenic callus development.

## Additional files

Additional file 1: Primers used in this study for qRT-PCR. (XLSX 14 kb)

Additional file 2: Lengths distribution of all assembled unigenes (XLSX 11 kb)

Additional file 3: Functional annotation of unigenes against Nr, Pfam, KEGG, COG, GO Swissprot databases. (XLS 20569 kb)

Additional file 4: Gene ontology (GO) classification, COG classification and KEGG classification of unigenes. a. The GO terms are summarized into three main categories: biological process, cellular component and molecular function. b. COG classification of the unigenes. c. KEGG classification of 5933 KO annotated unigenes. (XLSX 111 kb)

Additional file 5: Expression of genes in the 6 libraries in wheat callus based on the results of RNA-Seq. (XLSX 6574 kb)

Additional file 6: Information of DEGs in the comparisons of IME vs. ME. (XLSX 397 kb)

Additional file 7: Information of DEGs in the comparisons between stages in IMEs and MEs. (XLSX 319 kb)

Additional file 8: Functional annotation of all DEG against Nr, Pfam, Swissprot, KOG, KEGG, GO and COG databases. (XLSX 1510 kb)

Additional file 9: KEGG enrichment analysis of all DEGs. (XLSX 65 kb)

Additional file 10: List of all TFs and expression of DEGs TFs in the 6 libraries. (XLSX 93 kb)

Additional file 11: Expression and annotation of differentially expressed aux and SSEGs IME vs. ME. (XLSX 86 kb)

Additional file 12: KEGG pathways of plant hormone signal transduction in comparisons of IME vs. ME and between stages. (PDF 122 kb)

## Abbreviations

3 DC (6 DC; 15 DC): cultured for 3 d (6 d; 15 d)
ABA: Abscisic acid
ABI3: ABA insensitive 3
AGL: AGAMOUS-like
ARF: Auxin-responsive factor
BBM: Baby boom
BPGs: bio-processes-involved genes
CDPK: Calcium-dependent protein kinase
COG: Cluster of orthologous groups
DEGs: Differentially expressed genes
ERF: Ethylene-responsive transcription factor
GLP: Germin like protein
GO: Gene ontology
GST: Glutathione S-transferase
HSP: Heat-shock protein
IAA: Indole-3-aceticacid
IMEs: Immature embryos
KEGG: Kyoto encyclopedia of genes and genomes
LEA: Late embryogenesis abundant proteins
LEC: Leafy cotyledon
LTP: lipidtransfer protein
MEs: Mature embryos
RPKM: Reads per kilobase of exon model per million mapped reads
SE: Somatic embryogenesis
SSEGs: Stress and somatic embryogenesis related genes
TF: Transcription factor
XET: Xyloglucan endo-transglycosylase
XTH: Xyloglucan endo-transglucosylase/hydrolase protein
