# Supplementary figures and images for: De novo assembly and comparative analysis of the transcriptome of embryogenic callus formation in bread wheat (Triticum aestivum L.)
Source: BMC Plant Biol. 2017 Dec 19;17:244. doi: 10.1186/s12870-017-1204-2 (PMC5735865; doi:10.1186/s12870-017-1204-2)

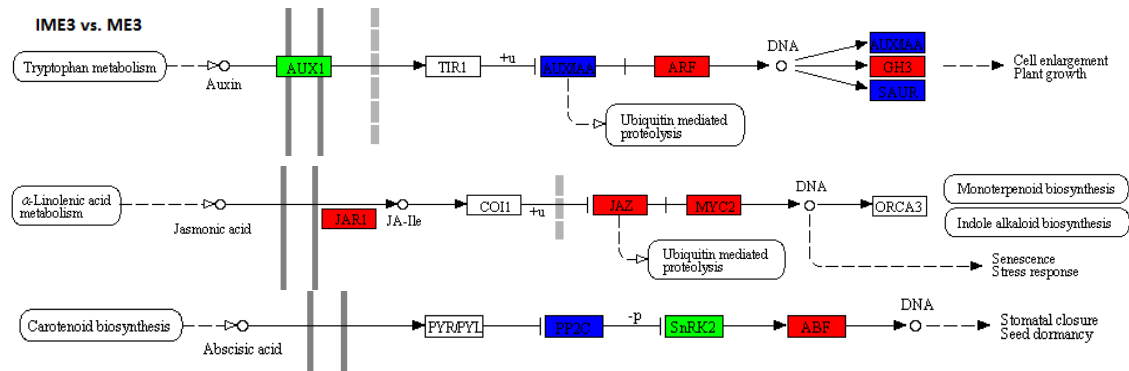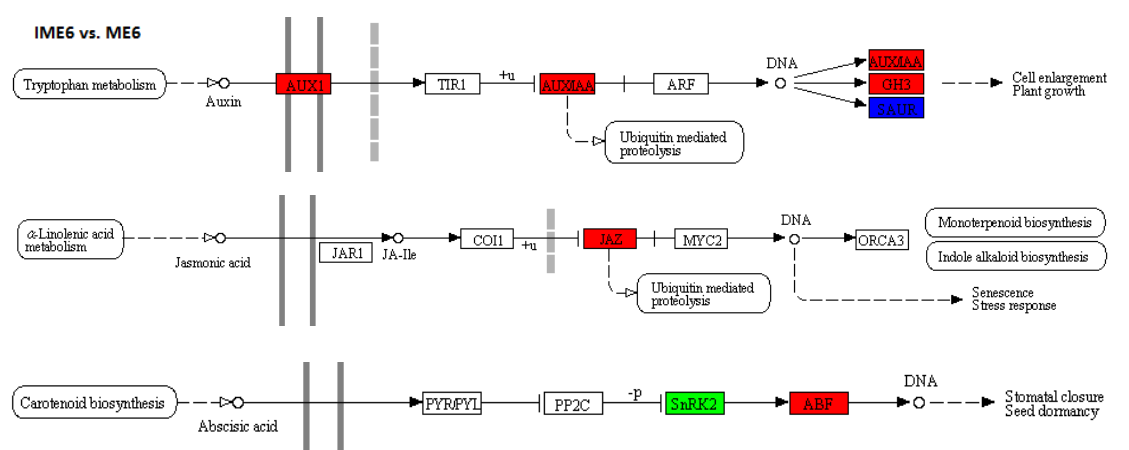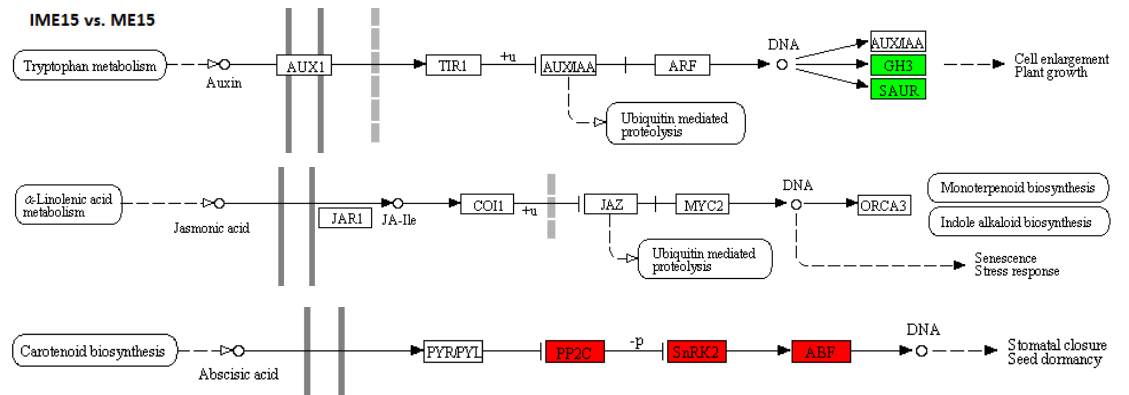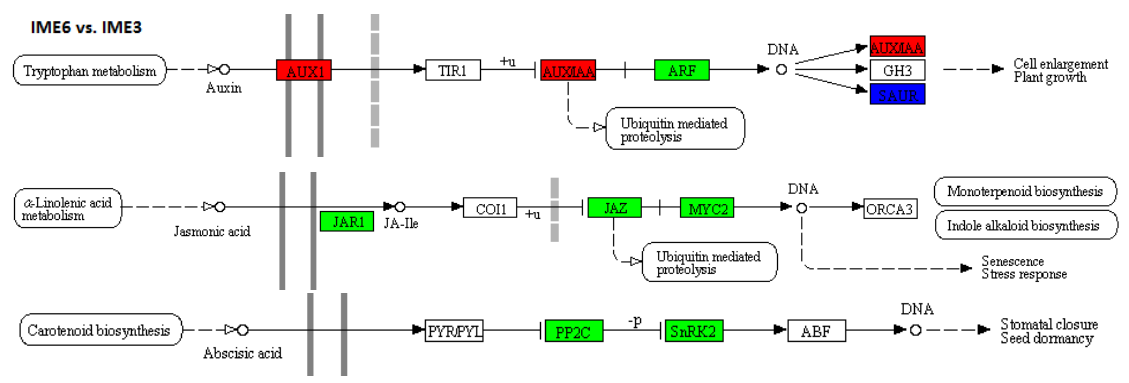

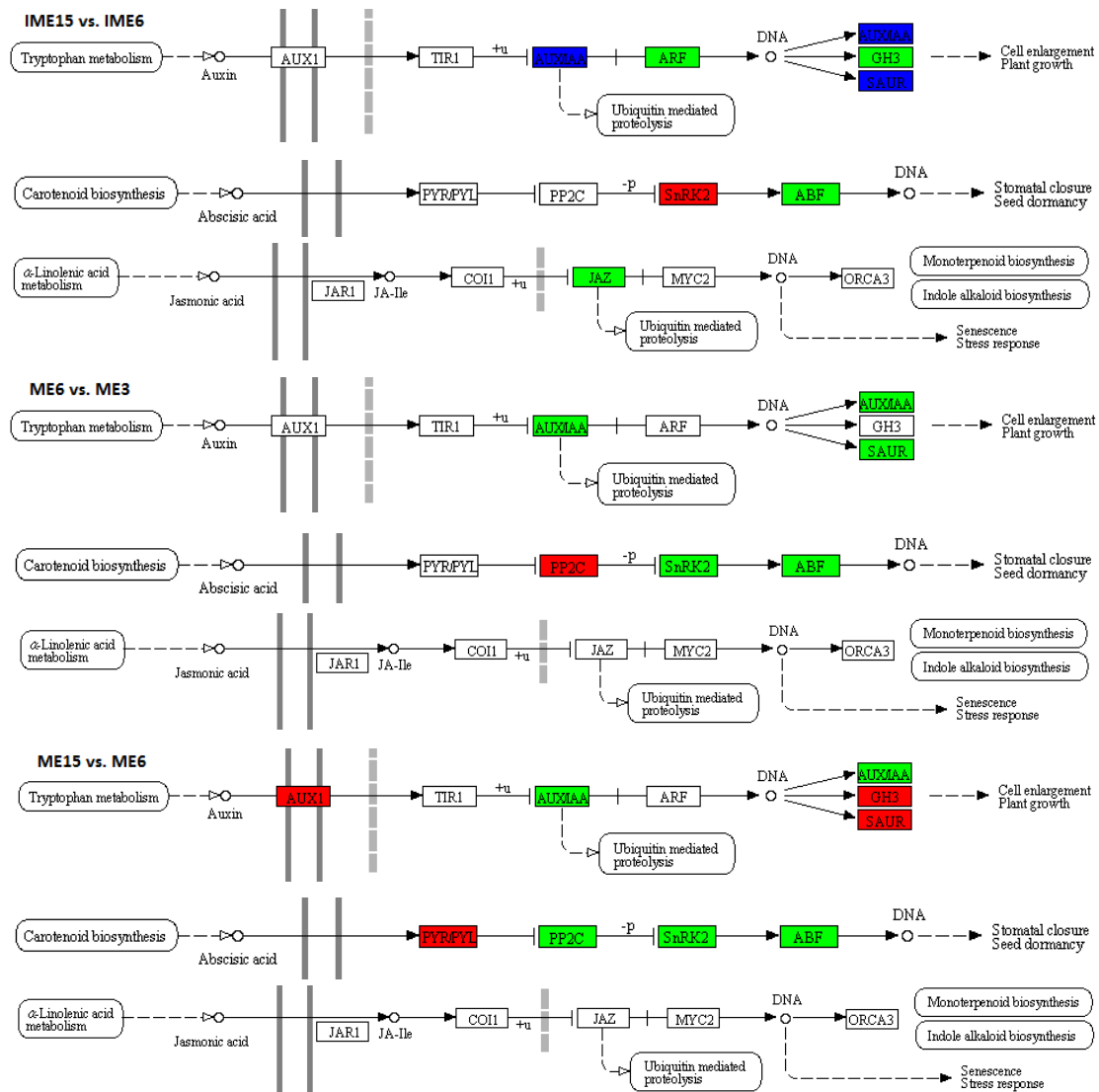

Supplement: Supplementary file 12 — KEGG pathways of plant hormone signal transduction in comparisons of IME vs. ME and between stages. (PDF 122 kb) [file 12870_2017_1204_MOESM12_ESM.pdf]
